# The Hidden Legacy of Dimethoate: Clay Binding Effects on Decreasing Long-Term Retention and Reducing Environmental Stability in Croatian Soils

**DOI:** 10.3390/toxics13030219

**Published:** 2025-03-17

**Authors:** Romano Karleuša, Jelena Marinić, Dijana Tomić Linšak, Igor Dubrović, Domagoj Antunović, Dalibor Broznić

**Affiliations:** 1 Department of Medical Chemistry, Biochemistry and Clinical Chemistry, Faculty of Medicine, University of Rijeka, Braće Branchetta 20, 51000 Rijeka, Croatia; romano.karleusa@uniri.hr (R.K.); jelena.marinic@uniri.hr (J.M.); domagoj.antunovic@uniri.hr (D.A.); 2 Department for Health Ecology, Faculty of Medicine, University of Rijeka, Braće Branchetta 20, 51000 Rijeka, Croatia; dijanatl@uniri.hr; 3 Department for Scientific and Teaching Activity, Teaching Institute of Public Health County of Primorje-Gorski Kotar, Krešimirova 52a, 51000 Rijeka, Croatia; 4 Department of Environmental Health, Teaching Institute of Public Health of Primorje-Gorski Kotar County, Krešimirova 52a, 51000 Rijeka, Croatia; igor.dubrovic@zzjzpgz.hr

**Keywords:** clay content, dimethoate sorption, metal ions, organic matter, organophosphate pesticides, pesticide stability

## Abstract

Understanding the dynamics of sorption and desorption is essential for assessing the persistence and mobility of pesticides. These processes continue to influence ecological outcomes even after pesticide use has ended, as demonstrated by our study on dimethoate behavior in distinct soil samples from Croatia, including coastal, lowland, and mountainous regions. This study focuses on the sorption/desorption behavior of dimethoate in soil, explores the relationship between its molecular structure and the properties of soil organic and inorganic matter, and evaluates the mechanisms of the sorption/desorption process. The behavior of dimethoate was analyzed using a batch method, and the results were modeled using nonlinear equilibrium models: Freundlich, Langmuir, and Temkin models. Soils with a higher organic matter content, especially total organic carbon (TOC), showed a better sorption capacity compared to soils with a lower TOC. This is probably due to the less flexible structures in the glassy phase, which, unlike the rubbery phase in high TOC soils, do not allow dynamic and flexible binding of dimethoate within the organic matter. The differences between the H/C and O/C ratios indicate that in high TOC soils, flexible aliphatic compounds, typical of a rubbery phase, retain dimethoate more effectively, whereas a higher content of oxygen-containing functional groups in low TOC soils provides strong association. The lettered soils showed stronger retention of dimethoate through interactions with clay minerals and metal cations such as Mg^2+^, suggesting that clay plays a significantly more important role in enhancing dimethoate sorption than organic matter. These results highlight the importance of organic matter, clay, and metal ions in the retention of dimethoate in soil, indicating the need for remediation methods for those pesticides that, although banned, have had a long history of use.

## 1. Introduction

Developed alongside organochlorines, organophosphate pesticides (OPPs) marked the advent of the modern pesticide era in the 1930s and 1940s. They enabled substantial increases in global food production while reducing the incidence of vector-borne diseases [1]. Due to their lower toxicity to mammals and lower persistence in the environment, organophosphates eventually replaced organochlorines [2] and became the most widely used broad-spectrum insecticides, accounting for 34% of global insecticide use [3]. Chlorpyrifos, chlorothalonil, dimethoate, phosmet, propiconazole and mancozeb are among plant protection products that are lost from the arsenal of OPPs, as unacceptable risks to human health and the environment have led to their banning or restriction in the EU and many industrialized countries [4,5,6,7].

Since OPPs are still widely used in Brazil, India, and other developing countries [8,9], and new OPP formulations [10] continue to be developed, it is expected that their use will continue to increase due to climate change [11]. Therefore, understanding their prevalence, their interactions with the environmental, and their long-term effects is crucial to reduce the future risks associated with OPPs and their derivatives [12].

In an arsenal of more than 100 different organophosphates, dimethoate [O,O-Dimethyl S-(N-methylcarbamoylmethyl) phosphorodithioate] (Table 1) is one of the insecticides and acaricides with systemic and contact effects that exhibits a cholinergic mechanism of toxicity centered on the inhibition of acetylcholinesterase (AChE) [13]. Due to its broad insecticidal spectrum, high effectiveness, and affordability, dimethoate has been one of the most widely used pesticides worldwide against a range of insects, including mites, flies, aphids, and plant hoppers, in both agricultural and urban areas [14]. It belongs to the class of phosphorodithioates, characterized by the presence of a pentavalent phosphorus atom with two single bonded methyl substituents, a double bond to a sulfur and a P-S single bond. A substituted monocarboxylic acid amide group loosely binding to the phosphorus atom through a single bonded sulfur atom is the “leaving group” that is eliminated upon phosphorylation of the OPPs, thereby inhibiting AChE [15]. This inhibition leads to an accumulation of acetylcholine, overstimulation of nicotinic cholinergic receptors, paralysis, and eventual death in both insects and mammals [16]. The no-observed-adverse-effect level (NOAEL) is determined for various organisms and routes of exposure based on doses that result in a 10–20% reduction in AChE activity in the brain or plasma [17].

Dimethoate exhibits varying toxicity across different organisms. It is slightly toxic to estuarine and marine invertebrates, highly toxic to freshwater fish and invertebrates, and very toxic to birds [18]. While dimethoate exerts its adverse effects primarily through inhibition of acetylcholinesterase, both acute and subchronic exposure have been linked to oxidative stress [19,20,21,22,23,24] and a potential for multisystemic toxicity. These effects include hepatotoxicity, nephrotoxicity, immunotoxicity, and toxic effects on the reproductive system, brain and pancreas [19,25,26]. According to the US Environmental Protection Agency (EPA), dimethoate is classified as a possible human carcinogen [27], but not according to the International Agency for Research on Cancer [28]. However, ongoing studies indicate that it may be carcinogenic and genotoxic in both in vitro and in vivo models [29,30].

Studies in various environmental media revealed that dimethoate undergoes hydrolysis and microbial degradation, with negligible photodegradation due to its stability under light exposure [14,31,32]. Hydrolysis, a major dissipation pathway, is pH-dependent—faster under alkaline conditions (12-day half-life at pH 9), but significantly slower in acidic to neutral soils (pH 2–7), where dimethoate remains more stable [33]. The half-life of dimethoate spans from 18 h to 8 weeks in water [34], and 2 to 5 days in plants [33]. In soil, its half-life ranges from 4 to 16 days [34], but can extend up to 206 days in the absence of biodegradation [35]. Although dimethoate requires relatively low effective doses (50–600 g/ha) to control various pests, repeated applications are often required throughout the growing season [36], which may further enhance its persistence in soil leading to accumulation of dimethoate in the environment [37,38] and in the human body [39] via the food chain.

The persistence of dimethoate in soil is closely related to its low log *K*_OW_ (Table 1) and high water solubility (Table 1), which increase its mobility and make it susceptible to leaching [40,41]. As a result, dimethoate residues have been detected in both surface and groundwater globally, with levels frequently surpassing the World Health Organization’s guideline limit of 6 µg/L for dimethoate in drinking water [42]. Notable cases include northern China [43], California [44], and the Mediterranean Sea, where concentrations in the surface water reached up to 39.9 µg/L [45]. Due to these concerns, dimethoate was initially listed on the USEPA CCL 3 list as a contaminant of concern for drinking water safety [46].

Beyond water contamination, dimethoate and its toxic oxy-metabolite, omethoate, have also been found in soil, as well as in fruits, vegetables, and dairy products [47]. Many of these commodities are deeply embedded in Europe’s socio-economic and cultural heritage, such as olive crops. Spain, Italy, and Greece, as leading olive oil producers, have reported significant dimethoate residues, with 33.3% of Greek and 44.6% of Italian conventional olive oil samples testing positive in the 2000s [48]. Agricultural activities near estuaries further increase the risk of dimethoate entering coastal waterways and impacting estuarine ecosystems [49], particularly in Mediterranean regions, where low soil organic matter (<2%) [50] reduces sorption efficiency, increasing potential for leaching. This has been demonstrated in studies of soils from olive plantations in the Croatian coastal region [51].

In addition to organic matter, the sorption of dimethoate in the soil matrix and its potential migration to groundwater are largely influenced by clay content and the soil’s cation exchange capacity, as several studies have shown [51,52,53,54,55,56]. Additionally, dimethoate contains polar functional groups (-NH, -C=O, and -P=O) that can form hydrogen bonds with soil organic matter or hydroxyl groups on the surface of clays [53,57,58]. Under acidic conditions (pH < 6), it can protonate, leading to interactions with negatively charged soil particles, such as silicate groups or humic acids [53,57]. As a result, the sorption of dimethoate into soil particles involves multiple processes, influenced by the physicochemical properties of both the soil and the dimethoate molecules. Despite dimethoate being among the highest-loading pesticides and one of the three most widely used active substances in the EU 26+1 (including Croatia) until its ban [59], the sorption behavior of dimethoate in specific soil types remains limited, leading to uncertainties in modeling its movement across diverse geological conditions and posing a challenge for comprehensive environmental management in the EU.

To address these issues, we have conducted sorption/desorption studies of dimethoate with three main objectives: (a) to investigate the sorption/desorption behavior of dimethoate in various soil types; (b) to examine the relationship between its molecular structure and molecular variations to the nature of soil organic matter; and (c) to model possible mechanisms driving the sorption/desorption process.

We believe that the findings of this study will help prevent the replacement of hazardous pesticides with structurally similar alternatives that may pose equal or greater risks. Furthermore, this research supports the broader objectives of the EU Soil Strategy for 2030 [60] and the proposed Soil Monitoring Law [61] by contributing to the protection, restoration, and sustainable management of soils across EU Member States.

**Table 1:** Chemical structure and physicochemical properties of dimethoate [62].

Chemical structure	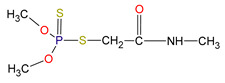	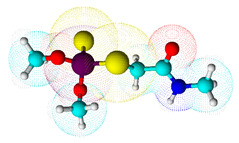
IUPAC name	*O*,*O*-Dimethyl S-(N-methylcarbamoylmethyl) phosphorodithioate	
Molecular formula	C_5_H_12_NO_3_PS_2_	
Molar mass (g/mol)	229.3	
Log *K*_OW_	0.704 (pH = 7; 20 °C)	
Soil sorption coefficient (*K*_OC_)	20	
Water solubility (mg/L)	39,800 (21 °C)	
p*K*_a_	no dissociation	
Hydrogen bond donor count	1	
Hydrogen bond acceptor count	5	
Topological polar surface area (Å^2^)	105	
DT50 in aqueous solutions (days)		
pH = 2–7	Stable	
pH = 9	12	

## 2. Materials and Methods

### 2.1. Chemicals

An analytical standard of dimethoate with a purity of >99.0% (Restek, Bellefonte, PA, USA) was used for the sorption–desorption experiments. Its physicochemical properties are listed in Table 1 [62]. To prepare the standard solution, dimethoate was dissolved in MS-grade methanol (Honeywell, Charlotte, NC, USA) and further diluted with methanol or the corresponding mobile phase. For the soil sorption experiment, a 1000 mg/L stock solution of dimethoate was prepared by dissolving 250 μL Chromgor^®^ 40 in acetonitrile ultra-gradient-grade (J.T. Baker, Deventer, The Netherlands). Working solutions with dimethoate concentrations ranging from 5.0 to 100.0 mg/L were then prepared by dilution with a 0.01 M calcium chloride solution (CaCl_2_, Acros Organics, Morris Plains, NJ, USA).

All solvents and chemicals were of analytical grade. These included: ammonium formate (Sigma-Aldrich, St. Louis, MO, USA), sodium acetate, sodium hydroxide, sodium pyrophosphate, potassium dichromate, sulfuric acid and phenolphthalein (Kemika d.d., Zagreb, Croatia). In addition, glucose (Merck, Darmstadt, Germany) and a certified EDTA standard (41.06 wt% C, 5.51 wt% H and 9.56 wt% N) were used (LECO Corporation, Saint Joseph, MI, USA). Deionized water was purified using a Siemens Ultra Clear system (Munich, Germany) to ensure high quality experimental conditions.

### 2.2. Soil Samples

**Figure 1:**
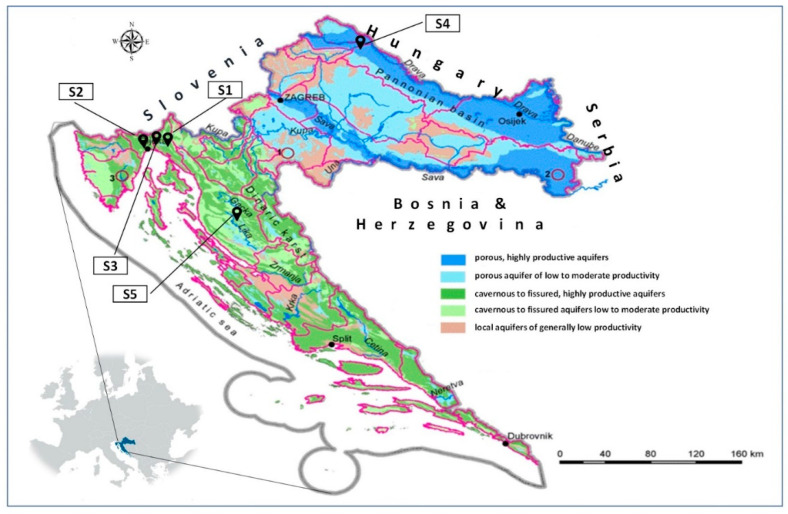
Locations of the soil samples (S1–S5) and their corresponding positions on the groundwater vulnerability map.

Sorption/desorption analyses of dimethoate were carried out on five soil samples in triplicate. Two samples were from the coastal area (Primorje-Gorski Kotar County), one from the lowlands (Varaždin County) and two from the mountainous area (Primorje-Gorski Kotar and Lika-Senj Counties) (Figure 1). The specific sampling locations and their geographical coordinates (Geographic Coordinate System, GCS) were: sample S1: mountainous region (Grobničko polje area, Čavle Municipality, Primorje-Gorski Kotar County), GCS: 45°20′53″ N, 14°30′04″ E; sample S2: coastal region (Matulji Municipality, Primorje-Gorski Kotar County), GCS: 45°21′27″ N, 14°18′20″ E; sample S3: coastal region (Matulji Municipality, Primorje-Gorski Kotar County), GCS: 45°21′28″ N, 14°18′19″ E; sample S4: plain region (Mali Bukovec Municipality, Varaždin County), GCS: 46°17′17″ N, 16°44′15″ E; sample S5: mountain region (city of Otočac, Lika-Senj County), GCS: 44°56′31″ N, 15°09′08″ E (Figure 1). These locations were selected to represent different soil types, providing an opportunity to assess how their characteristics affect the behavior of dimethoate. Given the intensive agricultural application, soil properties are of crucial importance. Soil samples were collected according to USEPA [63] standard soil sampling procedure. To ensure representativeness, five subsamples were taken from a depth of 0–30 cm and combined into a composite sample. This sample was quartered, air-dried, sieved through a 2 mm mesh, and stored in plastic containers at room temperature until analysis.

The collected soil samples were characterized by the following analyses: pH value (measured in a ratio of 1:2.5 soil/water and a 1:2.5 soil/0.01 M calcium chloride ratio), hydrolytic acidity—HA (Determined according to the Kappen method [64]); HA was calculated according the equation $\mathrm{HA}=\left(V\cdot k\cdot c\cdot 1000\right)/m$ (cmol/kg of the soil), where *V* is the volume of NaOH (mL), *k* is the factor of NaOH, *c* is the concentration of NaOH (mol/L), and *m* is the mass of soil (g); cation exchange capacity—CEC (determined according to the method of Hendershot et al. [65], the CEC was calculated using the following equation: $\mathrm{CEC}=\mathrm{HA}+\left[{\mathrm{Mg}}^{2+}\right]+\left[{\;\mathrm{Ca}}^{2+}\right]+\left[{\mathrm{Na}}^{+}\right]+\left[K^{+}\right]$ (cmol/kg of the soil), where the concentrations of cations were determined and calculated based on their respective atomic masses); organic matter (OM) content (analysed using potassium dichromate and concentrated sulfuric acid, with quantification on a UV/VIS spectrophotometer (UV–VIS Spectroquant^®^ Pharo 100, Merck, Darmstadt, Germany) [66]), humic (C_oxHA_) and fulvic acid (C_oxFA_) content determined by the method of Kononová and Belčiková [67]. The extracted humic acids were measured spectrophotometrically at wavelengths of 465 and 665 nm. After analysis, the obtained absorption values were used to calculate the E465/E665 ratio, which reflects the humic and fulvic acid content of the soil. Total organic carbon –TOC was analyzed according to HRN EN 15936 [68]. Samples were combusted in an oxygen stream at 900 °C, releasing CO_2_, which was quantified to calculate the carbon content. To remove inorganic carbon, a non-oxidizing mineral acid, such as HNO_3_, was used. The final TOC result was expressed as the percentage of carbon in the dry matter of the soil. A TOC module with a Non-Dispersive Infrared (NDIR) detector (Shimadzu TOC module, Kyoto, Japan) was used for TOC analysis. The content of carbon (C), hydrogen (H), nitrogen (N), sulfur (S) and oxygen (O) was analyzed according to HR EN 15407 [69] using a CHNS analyzer (LECO 628 CHNS, Saint Joseph, MI, USA). The analyzer was equipped with an Infrared (IR) detector for C and H, and a Thermal Conductivity (TC) detector for N. For the determination of sulfur (S), a special external module was required that works independently of the CHN analyzer. The sample was combusted in a ceramic boat under a stream of oxygen, resulting in the formation of SO_2_. The released gas was passed through a column directly into an IR cell and further analysis proceeded in the same way as for determination of the C and H content. The results for the C, H, N and S content were expressed as a percentage of each element in the dry mass of the soil. A calibration curve was prepared in triplicate using a standard solution of EDTA. The oxygen content (% O) was determined by calculations based on the elemental composition data. In addition, the atomic ratios H/C, C/N, S/C, O/C, and (N+O)/C were calculated based on the elemental composition using the relative atomic masses.

### 2.3. Sorption/Desorption Equilibrium Experiments

Dimethoate sorption/desorption tests were performed on triplicate soil samples using 50 mL polypropylene centrifuge tubes containing 5 g of soil and 25 mL of dimethoate solution at concentrations ranging from 5 to 100 mg/L [70]. A Unimax 1010 horizontal shaker (Heidolph, Schwabach, Germany) was used to equilibrate the suspensions h at 150 rpm and 20 (±1) °C for 24 h. After equilibration, the samples were centrifuged with a Rottina 420R centrifuge (Andreas Hettich GmbH & Co. KG, Tuttlingen, Germany) at 4500 rpm and 20 (±1) °C for 30 min. Prior to HPLC-MS/MS analysis, the supernatant was filtered using a syringe filter with a pore size of 0.20 μm (Filtres Fioroni, Senigallia, Italy). Control samples were used to exclude possible losses of dimethoate by sorption to the filter, on the walls of the centrifuge tube, or by evaporation, and included pesticide solutions without soil and soil samples without pesticide solution. Like the test samples, the two control sets were subjected to the same experimental and analytical processes. The observed loss of dimethoate from the initial concentration was calculated to be 1.5%, which was considered negligible.

The sorption of dimethoate, $q_{s}^{\mathrm{sor}}\left(\mathrm{eq}\right)$ (mg/kg), on the soils was calculated using Equation (1):

(1) $$q_{s}^{\mathrm{sor}}\left(\mathrm{eq}\right)=m_{s}^{\mathrm{sor}}\left(\mathrm{eq}\right)/m_{\mathrm{soil}}=\left[\gamma_{0}\;-\;\gamma_{\mathrm{aq}}^{\mathrm{sor}}\left(\mathrm{eq}\right)\right]\;\cdot V_{0}/m_{\mathrm{soil}}$$

$\gamma_{\mathrm{aq}}^{\mathrm{sor}}\left(\mathrm{eq}\right)$ denotes the mass concentration of dimethoate in the solution at equilibrium (mg/L), $m_{s}^{\mathrm{sor}}\left(\mathrm{eq}\right)$ is the mass of dimethoate sorbed in the soil at sorption equilibrium (mg), and $V_{0}$ is the initial volume of the dimethoate solution in contact with the soil (L).

After sorption, dimethoate equilibrium desorption experiments were carried out in the same soil samples. The aqueous dimethoate solution at equilibrium with the soil solid phase was removed and replaced with an equal volume (25 mL) of a 0.01 M CaCl_2_ solution. The soils were then resuspended in sterile H_2_O and homogenized using a vortex until they were uniformly mixed. They were then equilibrated for 24 h at experimental temperature on a rotary shaker. After this equilibration time, the soil suspensions were centrifuged, the supernatant filtered and analyzed for dimethoate content by the LC-MS/MS. The amount of dimethoate sorbed in the soil at desorption equilibrium was calculated according to Equation (2):

(2) $$q_{s}^{\mathrm{des}}\left(\mathrm{eq}\right)=(m_{s}^{\mathrm{sor}}\left(\mathrm{eq}\right)-m_{\mathrm{aq}}^{\mathrm{des}}\left(\mathrm{eq}\right))/m_{\mathrm{soil}}$$

Equations (3) and (4) were used to evaluate the total mass of dimethoate released from the soil at desorption equilibrium.

(3) $$m_{\mathrm{aq}}^{\mathrm{des}}\left(\mathrm{eq}\right)=m_{m}^{\mathrm{des}}\left(\mathrm{eq}\right)\cdot V_{0}/V_{r}^{F}\cdot m_{\mathrm{aq}}^{A}$$

(4) $$m_{\mathrm{aq}}^{A}\left(\mathrm{eq}\right)=m_{\mathrm{aq}}^{\mathrm{sor}}\left(\mathrm{eq}\right)\cdot \left(V_{0}-V_{R}\right)/V_{0}$$

In Equations (2)–(4), $q_{s}^{\mathrm{des}}\left(\mathrm{eq}\right)$ denotes the amount of dimethoate still sorbed in the soil at desorption equilibrium (mg/kg), while $\;m_{\mathrm{aq}}^{A}$, $m_{\mathrm{aq}}^{\mathrm{des}}\left(\mathrm{eq}\right)$ and $m_{m}^{\mathrm{des}}\left(\mathrm{eq}\right)$ represent the mass of dimethoate remaining in the soil due to incomplete volume compensation (mg), the total mass of insecticide desorbed from the soil at equilibrium (mg), and the mass of dimethoate in the aqueous phase at desorption equilibrium (mg), respectively. In addition, $V_{r}^{F}$ and $V_{R}$ represent the volume of solution removed for analysis at desorption equilibrium (mL), and the volume of supernatant removed after reaching sorption equilibrium and replaced by an equal volume of 0.01 M CaCl_2_ (mL), respectively.

### 2.4. Instrumentation and Operating Conditions

The concentration of selected cations: sodium (Na^+^), sodium (K^+^), calcium (Ca^2+^), and magnesium (Mg^2+^) were determined using an AAS800 Atomic Absorption Spectrometer (Perkin Elmer Analyst, Waltham, MA, USA), equipped with the AS 800 Autosampler (Perkin Elmer) and controlled by AAWinLab32 software (Perkin Elmer, Waltham, MA, USA). Cation analysis included digestion with concentrated HNO_3_, followed by microwave-assisted combustion using an MLS-1200 Mega Microwave Digestion System (Milestone, Sorisole, Italy) under the following conditions: 5 min at 300 W, 0.5 min at 0 W, 5 min at 600 W, and 1 min for ventilation. The samples were cooled, forming a homogeneous suspension further diluted with ultrapure demineralized water before AAS analysis. Calibration standards were prepared in a 0.1% HNO_3_ solution to match the conditions of the digested soil samples. Matrix effects were minimized by diluting the samples to concentrations at which interferences were considered negligible. Cation measurements were performed using flame atomization (FAAS). Each cation was quantified at its respective wavelength: Na^+^ at 589.0 nm, K^+^ at 766.5 nm, Ca^2+^ at 422.7 nm, and Mg^2+^ at 285.2 nm using the external standard method. The calibration curves (five concentrations in triplicate) showed that that linearity (*R*^2^ > 0.9950) was met in a range of 0.5–3 mg/L for Na^+^, 1–10 mg/L for Ca^2+^, 0.5–2 mg/L for Mg^2+^, and 0.5–2 mg/L for K^+^. The LOD and LOQ calculations were performed by the expressions: LOD = 3.3 × *r*/S and LOQ = 10 × *r*/S, where *r* is the standard deviation of the blank (determined from ten injections) and S is the slope of the calibration curve [71]. The calculated values resulted in LOQs for cations of 5 mg/kg each for Na^+^ and Mg^2+^, 10 mg/kg for Ca^2+^, and 0.5 mg/kg for K^+^. Each batch included a blank sample, quality control (QC) checked against a standard solution, and three parallel analyses per soil sample. The final results were expressed as mg/kg on a soil dry mass basis, ensuring reliable and reproducible measurements of cation concentration in soil samples.

Dimethoate residues were quantified using an LC-MS/MS system (Exion LC, SCIEX, Framingham, MA, USA) equipped with a Phenomenex Kinetex C18 analytical column (100 × 2.6 mm i.d. with precolumn, 2.6 µm particle size, 100 Å pore size, Phenomenex, Torrance, CA, USA). The mobile phases consisted of A (90% H_2_O, 10% CH_3_OH + 5 mM HCOONH_4_) and B (10% H_2_O, 90% CH_3_OH + 5 mM HCOONH_4_). The column was kept at 40 °C, and the automatic sampler was set to 4 °C. The sample injection volume was 10 µL, followed by gradient elution with the following conditions: 0–1 min, 98% mobile phase A and 2% mobile phase B; 15–18 min, 2% mobile phase A and 98% mobile phase B; 18.1–20.00 min, 98% mobile phase A and 2% mobile phase B. The total run time was 20 min, with dimethoate eluting at a retention time of 5.63 min.

### 2.5. MS/MS Conditions

Dimethoate was detected using a triple quadrupole mass spectrometer, SCIEX 4500 QTRAP, SCIEX (Framingham, MA, USA) coupled with an ESI source operating in positive ionization mode. The MS/MS instrument settings were: Ionization temperature 400 °C, ion spray voltage 4500 V, drying gas temperature 190 °C, drying gas flow 9.0 L/min. Nitrogen was used as nebulizing, curtain and collision gas. Multiple reaction monitoring (MRM) was used to monitor the two most intense precursor-product ion transitions for dimethoate, allowing for the selection of both quantifier and qualifier MRM transitions. Of the two fragments, the one with the higher intensity was used for quantification, while the other was used for confirmation. For dimethoate, an [M − H]^+^ precursor ion with a ratio m/z 230.0 was monitored in the quadrupole filter Q1, while the product ions 199.1 and 125.0 m/z were passed through the quadrupole filter Q3. The declustering potential was set to 41 V, the collision energy to 13.0 V and 29.0 V and the exit potential of the collision cell to 4.0 V. Data processing was carried out using Multiquant 3.6 software (SCIEX, Darmstadt, Germany). To minimize the risk of false positive results, a mass spectral library was used to identify compounds in Enhanced Product Ion (EPI) mode (AB Sciex, Framingham, MA, USA), thereby increasing confidence in the analytical results obtained.

The dimethoate calibration curve was linear within the concentration range of 0.1 to 10 mg/L, with a regression coefficient of *R*^2^ > 0.999. The analytical detection limit was 0.05 mg/L. The average recovery was 94.7%, with a relative standard deviation of less than 5%. A calibration curve was prepared before each batch of samples and verified using an insecticide standard at the beginning, middle, and end of each sample batch to ensure that there were no deviations in the intensity of the analyte. Samples with accuracy deviations of more than 20% were corrected. As no sample preparation (extraction) was required, the analysis was performed directly, and the accuracy of the previously prepared insecticide solution was confirmed. The calibration standards for dimethoate were prepared in a solution similar to the soil extracts to minimize matrix effects. Matrix effects were controlled using a matrix-matching approach, in which the standard solutions are prepared in a medium that simulates the conditions of the sample extracts. The samples were diluted by factors of 10 and 50 to a 1000 μL final volume to bring the concentrations of the analyzed insecticides into the validated measurement range.

### 2.6. Data Analysis

To analyze the sorption and desorption processes of dimethoate in the soil matrix, nonlinear isotherm models, the Freundlich, Langmuir, and Temkin models, were used.

The Freundlich isotherm is based on a heterogeneous surface, where the energy distribution on the surface on which the sorption of dimethoate can take place is discontinuous. It is represented mathematically by the Equation (5):

(5) $$q^{\mathrm{sor}/\mathrm{des}}=K_{F}^{\mathrm{sor}/\mathrm{des}}\gamma_{\mathrm{eq}}^{1/n}$$

In Equation (5), $K_{F}^{\mathrm{sor}/\mathrm{des}}$ is the Freundlich sorption/desorption coefficient (mg/kg) (mg/L)^1/n^, 1/*n* is the non-linearity coefficient, and $\gamma_{\mathrm{eq}}$ is the dimethoate equilibrium concentration in the solution (mg/L). The nonlinearity coefficient reflects the change in energy distribution on the heterogeneous soil surface and shows the different free energy levels required for the sorption of dimethoate on the different surface regions.

To evaluate the role of organic matter in the dimethoate sorption process, the organic carbon partition coefficient $K_{\mathrm{OC}}$ defined by Equation (6) was used:

(6) $$K_{\mathrm{OC}}=100\times K_{F}^{\mathrm{sor}}/f_{\mathrm{OC}}$$

In Equation (6), $f_{\mathrm{OC}}$ represents the percentage of OC in the soil.

In addition, the Gibbs molar free energy (ΔG) controls the partitioning of the pesticide between the solid and aqueous phases at equilibrium. The relationship between $K_{\mathrm{OC}}$ and ΔG is described by Equation (7):

(7) $$\Delta G=-\mathrm{RT}\ln K_{\mathrm{OC}}$$

The difference between sorption and desorption isotherms, or the comparison of the degree of hysteresis of dimethoate in different soils is expressed by hysteresis indices, *H* and *λ*. These indices were calculated using the following Equations (8) and (9) [72]:

(8) $$H=(1/n^{\mathrm{des}})/\left(1/n^{\mathrm{sor}}\right)$$

(9) $$\lambda =\left(\left(1/n^{\mathrm{sor}}+1\right)/\left(1/n^{\mathrm{des}}+1\right)\right)-1$$

In Equations (8) and (9), $1/n^{\mathrm{sor}}$ and $1/n^{\mathrm{des}}$ represent the previously defined Freundlich nonlinearity coefficients for sorption and desorption, respectively.

Langmuir’s isotherm model assumes that the sorption of dimethoate takes place on homogeneous soil surfaces in a monolayer, whereby the sorption capacity for dimethoate is finite and is denoted as $q_{\max}^{\mathrm{sor}}$. It is also assumed that all sorption sites are identical, sterically and energetically independent, and that each site sorbs only one molecule of dimethoate, which means that the amount of dimethoate sorbed at one site does not affect sorption on neighboring sites. The Langmuir isotherm model is described by Equation (10):

(10) $$q_{\mathrm{eq}}^{\mathrm{sor}/\mathrm{des}}=\left(q_{\max}^{\mathrm{sor}/\mathrm{des}}\;K_{L}^{\mathrm{sor}/\mathrm{des}}\;\gamma_{\mathrm{eq}}\;\right)/\left(1+K_{L}^{\mathrm{sor}/\mathrm{des}}\;\gamma_{\mathrm{eq}}\right)$$

According to Equation (10), $q_{\max}^{\mathrm{sor}/\mathrm{des}}$ is the maximum capacity of the soil to sorb dimethoate in a monolayer (mg/kg), and $\;K_{L}^{\mathrm{sor}/\mathrm{des}}$ is the Langmuir constant (L/kg), which refers to the enthalpy of sorption.

The Temkin isotherm model assumes that the sorption energy of all dimethoate molecules in the layer decreases linearly with surface coverage, reflecting the interactions between the dimethoate molecule and the soil matrix. It is also assumed that sorption occurs on heterogeneous surfaces with a uniform distribution of binding energy, with maximum binding energies reached as sorption progresses [73].

The model is represented by Equation (11):

(11) $$q_{\mathrm{eq}}^{\mathrm{sor}/\mathrm{des}}=\left(\mathrm{RT}/b\right)\ln\left(K_{T}^{\mathrm{sor}/\mathrm{des}}\gamma_{\mathrm{eq}}\right)$$

In Equation (11), $K_{T}^{\mathrm{sor}/\mathrm{des}}$ and b are Temkin equilibrium binding constant (L/mg) and the constant related to the heat of sorption, respectively, while R is the gas constant (8.314 J/mol·K), and T is the absolute temperature (K).

### 2.7. Statistical Analysis

The experimental data are presented statistically as mean values of triplicate analyses. Statistical comparisons were performed using Statistica^®^ 14.0.0 software (TIBCO Software, version 14.0.0, Palo Alto, CA, USA), with *p* < 0.05 assumed to be significant. The normality of the distribution of the data obtained was checked by applying the Kolmogorov–Smirnov test. A correlation matrix was used to determine the existing dependence between the investigated soil characteristic and the sorption/desorption parameters of dimethoate. Principal component analysis (PCA) was also carried out with the aim of highlighting the predominant physicochemical properties of the soil that significantly influence the process of dimethoate sorption/desorption. To further model the relationship between predictor variables and the parameters describing the process of dimethoate sorption/desorption, a multiple regression analysis was performed to propose predictive models for the sorption and desorption of dimethoate. The sorption/desorption parameters were estimated by nonlinear estimation using Wolfram Research Mathematica^®^ 11.0 software (Wolfram Research Co., version 11.0, Champaign, IL, USA). The accuracy of the models was checked by comparing the experimental data with the predicted values using the coefficient of multiple determination *R*^2^, Scaled Root Mean Squared Error (SRMSE), and *χ*^2^ test error.

## 3. Results and Discussion

### 3.1. Physicochemical Properties of Analyzed Soil Samples

Table 2 shows the physicochemical properties of the investigated soils. The soil texture varied from sandy loam (S2, 17.35% clay) with a limited retention capacity to a soil with high clay content in S4 (40.58%), which allows a greater retention of pesticides and nutrients. Soils S2 and S3 with less than 20.50% clay are likely to have lower sorption capacity, resulting in higher pesticide mobility and leaching risk. These results are consistent with studies on Croatian agricultural soils, where texture plays an important role in the retention of pesticides and nutrients [51,74,75]. Our findings, especially for soil S4, confirm previous studies that pesticide sorption is enhanced in soils with a high clay content due to stronger interactions between clay particles and pesticide molecules [51,55,76,77,78,79]. Soil pH ranged from 6.21 to 6.57, and HA was lowest at S4, (3.74 cmol/kg), while it was highest at S5 (27.51 cmol/kg). The CEC ranged from 66.12 cmol/kg for S5 to 91.32 cmol/kg for S2, indicating good nutrient retention. The highest TOC content was in S1 (5.75%) and the lowest in S4 (1.96%), indicating a lower organic matter content, which has the potential to control the structure of the soil. Based on the TOC content, the soils were low to moderately humic (1–5%), which is typical for Croatian arable soils [74,80,81,82]. The highest contents of fulvic (C_oxFa_, 0.118%) and humic acids (C_oxHa_, 0.312%) were found in S5. The hydrophilicity and hydrophobicity of the organic phase were determined using the H/C and O/C ratios. The highest H/C ratio (4.391) in S5 indicates the presence of more labile aliphatic compounds and the lowest ratio (S3) indicates the presence of more stable aromatic compounds. Higher H/C ratios favor the sorption of non-polar pesticides, but higher O/C ratios (S4; 46.436) in soils containing polar functional groups such as oxygen interact more effectively with polar pesticides. The C/N ratio varied slightly between the soils studied, with the highest values observed in soil S2 (13.594), while the lowest ratios were recorded in soil S4 (9.204). Soil S3 had the highest E465/E665 ratio of 6.25, which is attributed to its comparatively higher content of aliphatic compounds as compared to aromatic compounds and the presence of higher amounts of easily degradable aliphatic compounds. Sample S4 had the lowest ratio of 4.10, indicating a predominance of comparatively more stable aromatic compounds.

### 3.2. Evaluation of Dimethoate Sorption/Desorption in Croatian Soils Using Different Isotherm Models

The isotherm models of Freundlich, Langmuir and Temkin were used to simulate the sorption of dimethoate on Croatian soils (S1 to S5). Table 3 shows the results. The *R*^2^ values of the Freundlich model ranged from 0.9305 to 0.9773 with the lowest SRMSE and *χ*^2^-error percentage for S5 (0.0823 and 6.55%), indicating the best model fit. The highest *χ*^2^-error was found for soil S2 (16.10%). The *R*^2^ of the Langmuir model ranged from 0.7940 (S2) to 0.9753 (S4), indicating a moderate to strong fit. Compared to the Freundlich model, SRMSE and *χ*^2^-error were larger, and S2 had the largest *χ*^2^-error percentage (72.84%). The Temkin model did not fit soil S2 well, as evidenced by the lower *R*^2^ value of 0.6968 compared to S3 0.9080. Soil S2 had the lowest SRMSE values (0.5135), while soil S3 had the highest (0.2970). In addition, the percentage *χ*^2^-errors were higher than the Freundlich model, with soil S2 having the highest errors (40.83%).

According to the Freundlich model, all soils had moderate sorption capacity, with $K_{F}^{\mathrm{sor}}$ values ranging from 1.360 to 4.701 (mg/kg) (mg/L)^1/n^. S4 had the highest $K_{F}^{\mathrm{sor}}$ (4.701), indicating stronger sorption due to more active sorption sites, while S2 had the lowest value (1.360), indicating less effective sorption. These results are consistent with previous Croatian studies [51], which found that sorption coefficients ranged from 3.57 to 6.41 (mg/kg) (mg/L)^1/n^. Similar trends can be observed in comparisons with other regions. Higher OC and clay contents were associated with sorption coefficients ranging from 6.93 to 13.27 (mg/kg) (mg/L)^1/n^ in Australian soils [53]. With coefficients between 2.853 and 3.278 (mg/kg) (mg/L)^1/n^, Indian soils [58] exhibited lower sorption, comparable to our S2 soil. Soils in Tunisia [83] and Jordan [55] had sorption capacities of 2.11 (mg/kg) (mg/L)^1/n^ and 1.01 to 10.36 (mg/kg) (mg/L)^1/n^, respectively. Spanish soils [84] showed values between 1.0 and 1.7 (mg/kg) (mg/L)^1/n^, indicating weaker sorption, while our soil S4 showed higher retention. Mexican [85] and Spanish [86] soils varied between 1.06 and 8.94 (mg/kg) (mg/L)^1/n^, with Croatian soils, especially S4, showing comparable or higher sorption. According to Greek soils [57], Croatian soils have retention qualities comparable to other Mediterranean and semi-arid sites, with values ranging from 1.62 to 6.87 (mg/kg) (mg/L)^1/n^. With a range of 0.799 to 1.215 for the non-linearity coefficient (1/*n*), the soils investigated showed different sorption behavior. Soil S4 exhibited the most favorable sorption conditions with the lowest 1/*n* value (0.799), indicating increasing sorption at higher concentrations and effective sorption at low concentrations. Soils S2, S3, and S5 exhibited cooperative sorption with 1/*n* values above 1 (the highest value for S3 was 1.215). At higher dimethoate concentrations, this was most likely due to changes in soil–solution interactions or uniform surface properties. Soil S1 exhibited a uniform sorption energy over its surface, as shown by the almost linear sorption (1/n = 0.967). A comparison of the Croatian soils with the literature shows that they are relatively heterogeneous. A more heterogenous surface area was indicated by 1/*n* values between 0.66 and 0.81 obtained in our previous study [51]. A range of 0.95 to 1.38 was found for Australian soils [53], with some values higher than those found in our study. Like our S1 and S2 soils, the Indian soils [58] also had 1/*n* values ranging from 0.956 to 1.026. The range of 1/*n* for Jordanian soils [55] was 0.63 to 0.92, indicating strong to moderate sorption. With a low 1/*n* value of 0.49, Tunisian soils [83] showed high surface heterogeneity and significant affinity for dimethoate at low concentrations. The 1/*n* values of Greek [57] and Spanish [86] soils, which varied from 0.49 to 0.91 and 0.54 to 0.78, respectively, indicated high surface heterogeneity. The 1/*n* values of Mexican soils [85] were similar to those of our S4 soil and ranged from 0.74 to 0.88, confirming the role of OC and clay content in dimethoate retention. Spanish soils [84] had slightly higher values ranging from 0.88 to 0.93, similar to our S1 soil, indicating a more consistent sorption process. Compared to the Freundlich model, the Langmuir model showed greater variability, with $K_{L}^{\mathrm{sor}}$ values ranging from 0.0017 L/kg (S5) to 0.0263 L/kg (S4). Soil S1 exhibited the highest sorption affinity ($q_{\max}^{\mathrm{sor}}$ = 788.91 mg/kg), closely followed by soils S4 and S2. A poor fit to the Langmuir model was indicated by negative or low $q_{\max}^{\mathrm{sor}}$ values for S3 and S5, most likely due to multilayer interactions or heterogeneous sorption sites. In contrast, Mexican soils showed lower $q_{\max}^{\mathrm{sor}}$ values (3.49–4.02 mg/kg) but higher $K_{L}^{\mathrm{sor}}$ values (0.78–0.86 L/kg), indicating stronger binding despite lower retention [85]. Greek soils [57] showed moderate sorption with $q_{\max}^{\mathrm{sor}}$ between 11.87 and 25.51 mg/kg and $K_{L}^{\mathrm{sor}}$ between 0.17 and 0.53 L/kg. Australian soils showed very high $K_{L}^{\mathrm{sor}}$ values (80–310 L/kg) and $q_{\max}^{\mathrm{sor}}$ between 11.01 and 35.84 mg/kg, indicating strong sorption at low concentrations [53]. For the Temkin model, soil S4 had the highest $K_{T}^{\mathrm{sor}}$ value (0.3193 L/mg), while soil S2 had the lowest (0.1836 L/mg). The energy parameter $B_{1}^{\mathrm{sor}}$ was highest in S5 (71.98 J/mol) indicating stronger sorption energy, while the lowest value (37.59 J/mol) was observed in S4, indicating a more stable sorption. Compared to Indian soils [58], which had lower $B_{1}^{\mathrm{sor}}$ values (9.72–11.30 J/mol), and higher $K_{T}^{\mathrm{sor}}$ values (1.305–1.497 L/mg), Croatian soils exhibited a more balanced sorption profile. For most samples, especially those with low or negative $q_{\max}^{\mathrm{sor}}$ values, the assumption of monolayer sorption is insufficient, although the Langmuir model is appropriate for certain soils, like S4. Although the Temkin model had higher *χ*^2^-error percentages and lower *R*^2^ values for most soils, especially S2, it was useful for understanding sorption energy. In contrast, the Freundlich model showed continuously high *R*^2^ values, relatively low *χ*^2^ error percentages, and a low SRMSE. Due to its ability to account for surface heterogeneity and different sorption intensities, it is the most suitable model to understand the sorption of dimethoate.

The desorption of dimethoate in Croatian soils (S1 to S5) was analyzed with the same models—Freundlich, Langmuir, and Temkin—that were used in the sorption study. The statistical parameters and desorption characteristics for each soil group are shown in Table 4. The Freundlich model showed a high *R*^2^ (0.9912 for S1 to 0.9989 for S2) and a low SRMSE (0.0468 for S5), with a *χ*^2^-error ranging from 3.72% (S5) to 6.80% (S1). The *R*^2^ values of the Langmuir model ranged from 0.9523 (S5) to 0.9802 (S4), however, the *χ*^2^-error percentages were higher, with soil S5 having the largest error percentage (23.62%). The SRMSE for the Langmuir model showed variations in soils with increased desorption capacity and ranged from 0.1794 for S4 to 0.2969 for S5. The Temkin model showed the highest *χ*^2^-error (28.50% for S5) and the lowest *R*^2^ values (0.8733 for S2 to 0.9463 for S4).

The hysteresis in the retention process was indicated by the desorption coefficients ($K_{F}^{\mathrm{des}}$), which ranged from 3.482 (S2) to 9.096 (mg/kg) (mg/L)^1/n^ (S4). These coefficients were greater than the sorption coefficients. As observed in soil S4, the 1/*n* for desorption varied from 0.809 (S4) to 0.911 S2), with lower values favoring desorption. Compared to Croatian soils (S3 and S4), Indian soils [58] initially exhibited higher values, ranging from 9.528 to 12.410 (mg/kg) (mg/L)^1/n^, with 1/*n* between 0.747 and 0.825, and values similar to those of Croatian soils. In contrast, Tunisian soils [83] had $K_{F}^{\mathrm{des}}$ = 126.8 (mg/kg) (mg/L)^1/n^ and a 1/*n* = 0.65. In addition, $K_{F}^{\mathrm{des}}$ (40.58–66.57 (mg/kg) (mg/L)^1/n^) were higher in Australian soils [53]. In the Langmuir model, $K_{L}^{\mathrm{des}}$ varied between 0.0200 L/kg (S2) and 0.0760 L/kg (S4), with S5 having the highest retained dimethoate ($q_{\max}^{\mathrm{sor}}$) at 220.02 mg/kg and S4 having a lower one at 127.44 mg/kg. The Temkin model showed $K_{T}^{\mathrm{des}}$ ranging from 0.8934 L/mg (S2) to 1.3799 L/mg (S4), with $B_{1}^{\mathrm{des}}$ values from 16.85 (S2) to 25.51 (S5).

Despite the good fit to dimethoate desorption, the accuracy of the Langmuir model was poorer than that of the Freundlich model, as reflected by larger *χ*^2^-error percentages. With the highest errors and the lowest *R*^2^ values, the Temkin model performed the worst. The Freundlich model was therefore the most reliable, with high *R*^2^ values, low SRMSE, and low error percentages.

### 3.3. Thermodynamic Analysis of Dimethoate Sorption/Desorption in Croatian Soils

Some thermodynamic parameters of the sorption/desorption process of dimethoate, namely *K*_OC_, ΔG, and hysteresis coefficients, *H* and *λ* of the investigated Croatian soils are listed in Table 5. The values of *K*_OC_ are highly different among the soil samples. Dimethoate in S2 and S3 soils (37.09 L/kg and 33.36 L/kg, respectively) showed high mobility, as indicated by McCall: *K*_OC_ < 50 L/kg [87] is considered to be highly leached into deeper layers and a potential contaminant of groundwater. Soils S1 and S5 had *K*_OC_ values of 53.63 L/kg and 58.57 L/kg, respectively, classifying them as soils with moderate mobility. A *K*_OC_ values between 50 and 150 L/kg indicates a slightly lower, but still significant risk of leaching. In contrast, soil S4 (240.49 L/kg) shows a moderate mobility of dimethoate (*K*_OC_ between 150 and 500 L/kg), indicating a higher retention and a lower leaching potential compared to the other soils. In contrast to the previous findings, studies from other regions showed varying degrees of dimethoate mobility. The mobility in Mexican soil was high, *K*_OC_ < 50 L/kg [85], while in Australian and Greek soils the *K*_OC_ were highly variable, ranging between 163 and 5023 L/kg in Australian soil [53] and 60.19 and 663.43 L/kg in Greek soil [57]. In Tunisian soils [83], the *K*_OC_ values ranged from 129 to 184 L/kg, while the value in Japanese soils [52] was 82.4 L/kg. In Indian soils, dimethoate showed moderate mobility, with *K*_OC_ > 500 L/kg [58]. Spanish soils displayed moderate to high mobility (*K*_OC_ ranging from 50 to 192 L/kg), indicating a considerable leaching potential [84].

The negative values of ΔG in all soils, ranging from −8.48 kJ/mol to −13.36 kJ/mol, confirmed that the sorption of dimethoate was spontaneous, as reported in previous studies [57,58]. In general, a negative value of ΔG indicates spontaneity in the sorption process, such that dimethoate readily sorbed to the soil particles, especially in soils with higher organic matter content [57,58]. Indeed, such negative ΔG values for the dimethoate sorption in Croatian soils were also obtained in our previous study, clearly emphasizing the importance of OC and clay content for dimethoate retention [51]. The values showed a variation between soils, with soil S4 having the most negative ΔG (−13.36 kJ/mol), indicating a strong sorption of dimethoate in this soil. In contrast, soils S2 and S3 with less negative ΔG values of −8.79 kJ/mol and −8.48 kJ/mol, respectively, showed weaker sorption and higher leaching potential. The ΔG values are generally used to differentiate between physisorption (weak, reversible interactions) and chemisorption (strong, irreversible interactions). Physisorption generally occurs at ΔG values between −5 and −10 kJ/mol, whereas chemisorption is characterized by values greater than −20 kJ/mol [88]. In this study, the ΔG values (−8.48 to −13.36 kJ/mol) indicate that the sorption of dimethoate occurs primarily through physisorption, with minor contributions from chemically enhanced interactions, especially in soils with higher OC content.

The hysteresis coefficients, *H* and *λ*, provide further information on the reversibility of the sorption and desorption processes. The highest *H* value of 1.012 was observed in soil S4, indicating strong hysteresis and limited reversibility of dimethoate desorption, which is consistent with the high *K*_OC_ and strongly negative ΔG obtained for this soil. Soils S3 and S5, with the lowest *H* values (0.713 and 0.750) showed easier desorption of dimethoate from these soils. The coefficient *λ* reflects the reversibility of sorption, while a negative value of *λ* for soil S4 (−0.005) indicates a more asymmetric or slower desorption process. Our results are higher compared to the values reported in the literature than those observed in Indian soils, where the value of *H* ranged between 0.747 and 0.825 [58]. Similar trends were also observed in studies with soils from Australia [53], which showed *H* values between 0.72 and 0.90. These differences indicate that greater hysteresis and reversibility are limited in Croatian soils, especially in soil S4, compared to Indian and Australian soils.

### 3.4. Dimethoate Sorption/Desorption in Soils: Freundlich Isotherms and the Influence of Soil Physicochemical Properties and Dimethoate Concentration on Curve Shapes

**Figure 2:**
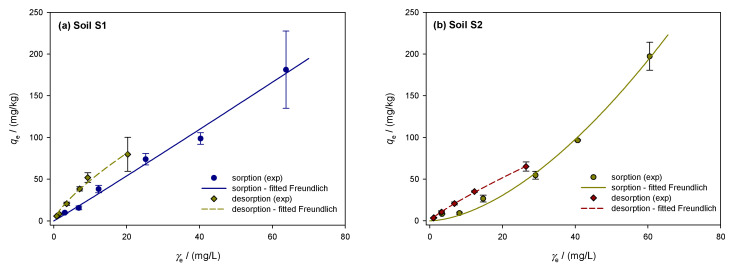

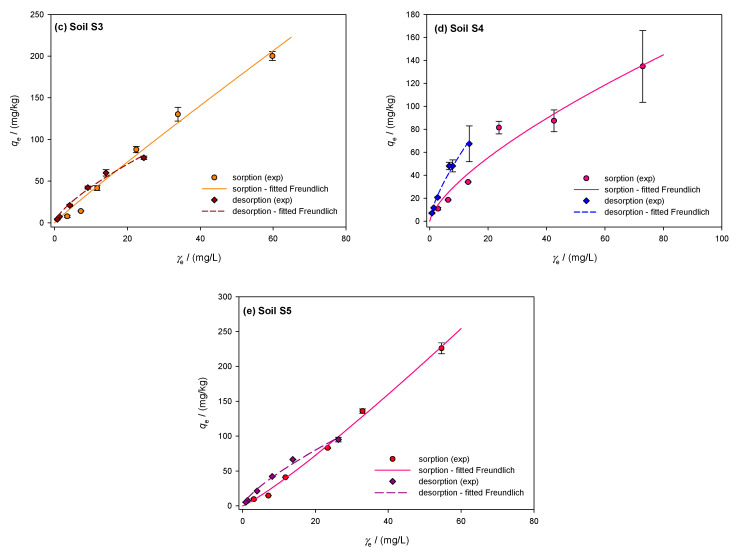
Freundlich isotherms showing the sorption and desorption of dimethoate in the investigated soils S1–S5 (**a**–**e**): A comparative analysis of experimental data and theoretical nonlinear model fits, where the symbols represent the experimental data and the lines represent the predictions of the Freundlich equilibrium model.

The sorption/desorption isotherms of the investigated soils were described using the Freundlich model. Figure 2a–e shows the sorption isotherms of the soils, represented by the solid line. Sorption in clay loam soil S1 averaged 35.8 ± 3.1% with a coefficient of variation of 8.7% and was constant across all dimethoate concentrations. The sorption capacity was the highest at 5 mg/L, (39.2%), and lowest at 10 mg/L (31.3%). The high OM content (TOC = 5.75%) and the CEC (74.39 cmol/kg) indicate strong pesticide binding. According to Valverde-García et al. [86], there is a relationship between sorption and soil properties, such as organic matter and CEC, as shown by the almost linear Freundlich isotherm (1/*n* = 0.967). Although dimethoate showed an “S”-type isotherm consistent with the findings in Indian soils, the L-type curves indicate a high initial affinity between the solid surface and dimethoate solution [21,55]. L-type isotherms were also observed in other studies by Al Kuisi [55] and Van Scoy et al. [14], indicating a strong sorption affinity possibly caused by a higher clay content. Limited sorption sites on the colloidal surface of the soil competing with the solvent indicate initially modest sorption concentrations. For non-ionizable pesticides such as dimethoate, the tendency of pesticide molecules to bind by hydrophobic contact increases with sorption [55]. According to Hernandez-Soriano et al. [88], organic amendments had no effect on the retention of dimethoate, suggesting that organic matter plays a modest role. However, the amount of organic matter present seems to have an influence on sorption [55], especially in Jordanian soils. With a coefficient of variation of 24.2% and an average value of 29.5 ± 7.1%, the sandy loam soil S2 exhibited greater variance and less pronounced sorption. At 10 mg/L, the sorption efficiency decreased from 39.5% to 18.4%. The lower clay content (17.35%) and OM (TOC = 3.68%) resulted in less active sorption sites, as shown by the convex Freundlich isotherm (1/*n* = 1.143). This behavior agrees with the results of Rani et al. [58], who observed S-shaped isotherms in soils with low organic matter and clay content. The sorption efficiency of sandy clay loam soil S3 decreased from 28.0% to 30.8% at lower concentrations (10 mg/L) but remained constant at higher concentrations (40.2% at 100 mg/L, 43.9% at 20 mg/L). The convex Freundlich isotherm (1/*n* = 1.215) showed a slower decrease at lower concentrations and a stronger sorption at higher concentrations. This agrees with the results of Van Bladel and Moreale [89], who found that isotherms shift from L-type to S-type with an increasing amount of organic matter. Similar to the results of this study, Vagi et al. [57] also found that dimethoate isotherms shift from S-type to L-type with increasing organic matter content in the soils. The average sorption capacity of clay soil S4 was 35.3 ± 6.4%, with a coefficient of variability of 18.3%. Sorption was greater at lower concentrations, reaching 43.5% at 5 mg/L. Sorption decreased at higher concentrations (100 and 60 mg/L), reaching 27.1 and 29.1%, respectively. The effective retention of dimethoate in soil S4 was favored by the high clay content (40.58%) and CEC (89.37 cmol/kg). The concave Freundlich isotherm (1/*n* of 0.799) showed stronger retention at higher concentrations, while sorption efficiency gradually decreased at lower concentrations. This is consistent with studies by Broznić et al. [51], who found that L-type isotherms, indicating a strong affinity for dimethoate, were associated with high clay content and organic matter content. Soil S5 had a coefficient of variation of 14.7% and a mean sorption capacity of 40.2 ± 5.9%. At lower concentrations, the sorption efficiency decreased significantly and peaked at 100 mg/L (45.4%). At lower concentrations, the convex Freundlich isotherm (1/*n* = 1.166) showed a sharp decrease in sorption efficiency. This pattern is consistent with Sharma et al. [21], who observed that soils with lower clay and organic matter content showed greater variations in sorption efficiency. With increasing equilibrium concentration, Valverde-García et al. [86] observed a significant increase in sorbed dimethoate, with sorption at 10 mg/L being four to five times higher than at 1 mg/L.

The dimethoate desorption isotherms for the investigated soils are shown in Figure 2a–e with dashed lines and show the amount of dimethoate remaining after the desorption process (mg/kg) vs. the equilibrium desorption concentration (mg/L). The percentage of desorbed dimethoate is calculated by dividing the amount desorbed from the soil by the total amount sorbed. With a moderate coefficient of variability of 9.7% and an average desorbed amount of 47.7 ± 4.6% in soil S1, the desorption process appears to have been fairly constant. The lowest desorption was 42% at 5 mg/L, while the highest was 56% at 100 mg/L. A concave desorption isotherm, indicated by the linearity coefficient of 1/*n* = 0.866, shows that the efficiency decreases with decreasing concentration. Soil S2 showed a decrease in desorption intensity from 67% at 100 mg/L to 58.9% at 5 mg/L, with a small variability (4.7%) and a larger desorption of 62.1 ± 2.9%. Since the desorption intensity is relatively constant across concentrations, the 1/*n* value of 0.911 indicates that soil S2 does not sorb dimethoate efficiently. Soil S3 had a mean desorption of 52.0 ± 5.1% and greater variability (9.8%) than S2. Desorption decreased from 61% at 100 mg/L to 47% at 5 mg/L. The concave isotherm behaved similarly to S1 and S2 with a 1/*n* of 0.863. Soil S4 showed the lowest desorption, with an average desorption of 41.3%, a coefficient of variability of 13.3%, and a standard deviation of 5.5%. The desorption intensity decreased from 50% at 100 mg/L to 35% at 5 mg/L. The concave isotherm for soil S4 shows that it retains dimethoate more effectively at higher concentrations, but that retention decreases at lower concentrations, with a 1/*n* value of 0.809. In soil S5, desorption reached its maximum at 100 mg/L (58%) and decreased to 45% at 5 mg/L. The average desorption was 49.5%, with a standard deviation of 4.7% and a moderate coefficient of variability of 9.5%. Similar to soil S1, the isotherm shape in S5 was concave, with a value of 1/*n* = 0.872. The mass balances of dimethoate showed a significant amount of free insecticide, which increased with temperature and initial concentration according to Vagi et al. [57]. The high percentages of desorption observed in soils S2 and S3 are in agreement with these results. Rani and Sud [58] found that up to 60% of dimethoate remained free in solution during sorption at 20 °C, and this percentage increased with increasing temperature. Dimethoate was released into the water to a greater extent as desorption was significantly affected by higher temperatures and initial concentrations.

### 3.5. Correlations Between Soil Characteristics and Dimethoate Sorption/Desorption Processes

The sorption and desorption behavior of dimethoate is strongly influenced by the properties of the soil, especially by the organic matter and the inorganic phases. The influence of soil physical and chemical properties on key sorption and desorption parameters, including $K_{F}^{\mathrm{sor}}$, 1/*n*^sor^, $K_{F}^{\mathrm{sor}}$, 1/*n*^des^, *K*_OC_, ΔG, and hysteresis indices (*H* and *λ*), was investigated using correlation analysis. The results are shown in Table 6.

The negative correlation between soil pH and 1/*n*^sor^ of −0.59 (*p* = 0.021) indicates that dimethoate retention decreases with increasing pH. Vagi et al. [57] demonstrated that higher pH levels lead to a reduction in active sorption sites and less nonlinear sorption. The positive correlation between pH and hysteresis coefficient *H* (0.58, *p* = 0.024) indicates better long-term retention at higher pH. The pH also negatively correlated with *λ* (−0.61 *p* = 0.017), which affects both the sorption rate and the long-term mobility of pesticide. This is consistent with Islam et al. [90], where higher pH increased leaching and mobility. Sorption ($K_{F}^{\mathrm{sor}}$) showed strong positive correlations with organic matter (0.96) and clay content (0.91), while Kuisi [55] observed negative correlations with pH (−0.79). The HA affected sorption by increasing the nonlinear nature of sorption (1/*n*^sor^ = 0.61, *p* = 0.016), while negative correlations with *K*_OC_ (−0.57, *p* = 0.026) and hysteresis coefficient *H* (−0.59, *p* = 0.014) suggest that HA decreases long-term retention of pesticides, as observed by Garg et al. [91] and Ismail et al. [92]. In our study, the CEC showed poor correlation with the sorption and desorption of dimethoate. Valverde-García et al. [86] determined by correlation analysis that the sorption capacity of dimethoate was most strongly correlated with soil specific surface area (*R*^2^ = 0.97, *p* < 0.001) and CEC (*R*^2^ = 0.94, *p* < 0.001). In addition, a weaker but still significant correlation was observed between dimethoate sorption and soil organic matter (*R*^2^ = 0.71) and clay content (*R*^2^ = 0.58). However, the processes were influenced by metals such as Mg^2+^ and K^+^; Mg^2+^ showed a weak positive correlation with sorption (0.52, *p* = 0.049), which contributed to the retention of dimethoate. This is consistent with the results of Rani and Sud [58], who also found a moderate positive correlation between the amount of Mg^2+^ and dimethoate retention. Conversely, an inverse relationship was observed between dimethoate desorption and K^+^ (−0.62, *p* = 0.014), suggesting that a higher K^+^ concentration in the soil promotes dimethoate retention by reducing its desorption. Similar results were reported by Islam et al. [91], who found that K^+^ reduced the mobility of pesticides in soils. According to Ismail et al. [92], Na^+^ had a negative effect on long-term retention (−0.57, *p* = 0.028), but Ca^2+^ had no effect.

Clay content was an important factor for both sorption (0.92, *p* < 0.001) and desorption, with a higher clay concentration reducing the leaching of pesticides. These results are in agreement with those of Garg et al. [91] and Valverde-Garcia et al. [86]. In addition, clay showed a positive correlation with *K*_OC_ (0.76, *p* = 0.001) and negative one with ΔG (−0.82, *p* < 0.001), suggesting that strong clay–pesticide binding reduces release to the environment by improving thermodynamic stability. Dimethoate was also stabilized by a strong negative correlation between clay concentration and the hysteresis coefficient *H* (−0.76 *p* = 0.001). This fact is supported by previous work by Islam et al. [91], who also found that high clay content improved pesticide retention and reduced leaching.

TOC content was inversely related to *K*_OC_ (−0.78, *p* = 0.001) and positively related with ∆G (0.75, *p* = 0.001), suggesting that higher TOC content increases the thermodynamic favorability of pesticide binding but has less influence than clay. Pesticide behavior was similarly influenced by C_OXFa_ and C_OXHa_ acids; C_OXFa_ showed a negative correlation with both sorption (−0.58, *p* = 0.022) and desorption (−0.52, *p* = 0.045), suggesting reduced retention. Moreover, C_OXFa_ increased λ (0.57 at *p* = 0.027), indicating increased retention. However, the C_OXHa_ and the *H* coefficient showed negative correlation (−0.59, *p* = 0.022), indicating a further decrease in long-term retention.

Elemental ratios, such as E465/E665, used as an index of soil organic matter structural characteristics, correlated significantly with both sorption and desorption of dimethoate. This ratio correlated negatively with sorption (−0.79, *p* < 0.001), and desorption (−0.57, *p* = 0.026), suggesting that a higher humic acid content improves retention by reducing mobility. The positive correlation with 1/*n*^sor^ (0.86, *p* < 0.001) indicates increased nonlinearity in sorption, while the inverse relationship with *K*_OC_ (−0.73, *p* = 0.002) and the positive correlation with ΔG (0.81, *p* < 0.001) further strengthen the role of humic acids in retention. In addition, a strong positive correlation with *λ* (0.90, *p* < 0.001) was observed. The C/N ratio negatively correlated with sorption (−0.89, *p* < 0.001) and *K*_OC_ (−0.89, *p* < 0.001), indicating that nitrogen-rich organic matter reduces retention. The negative correlation with desorption (−0.96, *p* < 0.001) indicates lower retention capacity, while the positive correlations with ΔG (0.92, *p* < 0.001) and *λ* (0.64, *p* = 0.011) imply increased environmental stability. Similarly, the O/C ratio correlated positively with sorption (0.73, *p* = 0.002), desorption (0.68, *p* = 0.006) and *K*_OC_ (0.95 at *p* < 0.001), suggesting stronger binding and long-term interactions. However, the negative correlations with ΔG (−0.90, *p* < 0.001) and *λ* (−0.64, *p* = 0.010), indicate lower Gibbs free energy and long-term retention. Finally, the S/C ratio correlated negatively with desorption (−0.52, *p* = 0.046), implying that sulfur-rich soils improve binding and decrease mobility of dimethoate.

### 3.6. Principal Component Analysis of Soil Characteristics Impacting Dimethoate Sorption and Desorption

**Figure 3:**
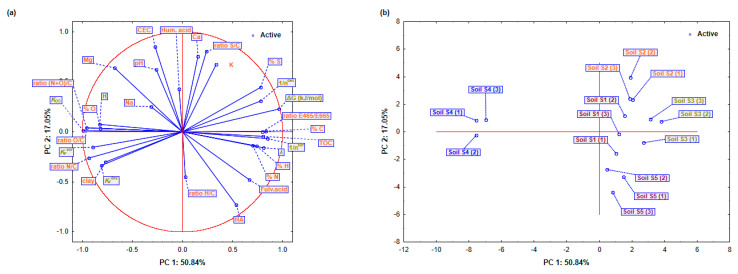
Comprehensive Principal Component Analysis (PCA) of physicochemical soil properties and Freundlich sorption/desorption parameters for dimethoate in soils (S1–S5): (**a**) Detailed projections of active variables (sorption/desorption parameters) and (**b**) soil properties and cases (soils) on the factor-plane (PC1 and PC2).

Principal component analysis was performed to simplify the data and identify significant features influencing the activity of dimethoate in soil. The four principal components (PCs), which explained 91.36% of the variance, showed the relationship between soil properties and dimethoate dynamics. The interpretation was based on the loading coefficients given in Table 7 and Figure 3a,b.

PC1 (50.84% variance) highlighted the variations in soil organic matter. TOC (0.8039 played a crucial role in dimethoate dynamics as it provides binding sites. Elemental components such as C (0.831), H (0.741), and S (0.776) further emphasized the importance of organic matter. The O/C ratio and PC1 show a negative correlation (−0.952), which means that oxygen-containing groups reduce the stability of dimethoate. The O/C ratio and PC1 show a negative correlation (−0.952), indicating that acidic groups reduce the stability of dimethoate, while ΔG (0.963) and *K*_OC_ (−0.991) indicate that organic matter stabilizes dimethoate and reduces leaching and mobility. Higher organic matter content decreases sorption, as shown by $K_{F}^{\mathrm{sor}}$ (−0.894), resulting in a change in the main retention mechanism towards mineral composition, such as clay (−0.772). PC2 was associated with soil texture and mineral properties (17.05% variance). CEC (0.850) showed strong ionic interactions in dimethoate retention. The importance of mineral composition is supported by high loadings for the S/C ratio (0.803) and Ca^2+^ concentration (0.756). The negative contribution of fulvic acids (−0.479) suggests that they may increase the mobility of dimethoate by influencing the availability of chelate metals and binding sites, especially with pH fluctuations. The influence of soil minerals was observed in PC3 (13.96% variance). Mg^2+^ (0.638) and K^+^ (0.673) increased retention by interacting with dimethoate molecules or mineral surfaces. However, Ca^2+^ (−0.305) could decrease the number of available binding sites by affecting soil aggregation. The positive N loading (0.634) suggests that nitrogen-rich materials support dimethoate retention, while $K_{F}^{\mathrm{sor}}$ (0.388) confirms that minerals also contribute to dimethoate sorption. The inorganic and organic compounds were related to PC4 (9.51% variance). Sorption increased with the H/C ratio (0.804) and humic acids (0.524), probably due to stronger organic binding. Desorption was affected by $K_{F}^{\mathrm{des}}$ (−0.291), indicating that the release of dimethoate is determined by both organic and mineral components. Ca^2+^ (−0.518) facilitated desorption by increasing mobility and weakening the bonds between dimethoate and soil particles.

A further analysis of the sorption/desorption behavior revealed significant differences in the dimethoate interactions between the soils (Figure 3a,b). In soils S1 and S5, the relatively high PC1 values showed a balance between organic and mineral properties. With a relatively high CEC (0.850) that supported stable sorption dynamics, and a TOC (0.803) that provided an abundance of binding sites, these soils provided consistent dimethoate retention. The negative loading of clay content (−0.772) suggests that organic matter plays a dominant role in reducing mobility and leaching risk. This implies that the organo-mineral properties are balanced to provide a good retention capacity for dimethoate, which reduces mobility and risk of leaching. On the other hand, the high TOC and clay content in soils S2 and S3 led to the remarkably favorable results for PC1 and PC2, improving the retention of dimethoate. CEC (0.850) enhanced the crucial role of TOC (0.803) by promoting ionic interactions that further stabilized dimethoate. Fulvic acids (−0.479), on the other hand, increased complexity by enhancing desorption under certain conditions, such as pH changes, thus increasing dimethoate bioavailability and runoff potential. Soil S4 showed a clear pattern with strongly negative PC1 values indicating low TOC levels and possible pH imbalances that hindered retention. With fewer organic binding sites (TOC 0.803) and weaker mineral interactions, desorption became more likely. Although this soil had the highest clay content, which could theoretically promote retention, its ability to sorb dimethoate decreased under neutral to high pH conditions. At elevated pH, interactions with humic acids and minerals were weakened, resulting in increased mobility.

### 3.7. Evaluation of Dimethoate Sorption and Desorption in Soils Using Multiple Regression: Statistical Modelling and Predictors Effects

Following the correlation and PCA analysis, a multiple regression analysis was performed to develop a predictive model for the sorption and desorption of dimethoate that provides deeper insights into its behavior in the analyzed soils. The results of the sorption model, presented in Table 8, show a very high *R*^2^ value of 0.9891, which means that 99% of the variance in dimethoate sorption is explained by the selected predictors. A high adjusted *R*^2^ value of 0.9782 confirms the strong agreement between experimental and model results, indicating that the selected variables are highly relevant, and the model is not overly complex. A high F-value of 90.56 and a very small *p*-value (less than 0.0001) emphasize the statistical significance of the model and show that at least one predictor significantly influences sorption. Among the important predictors for the $K_{F}^{\mathrm{sor}}$ coefficient, clay was found to have a positive effect (0.364, *p* = 0.015), suggesting that a higher clay content improves the soil’s ability to retain dimethoate. Several variables had a negative effect on sorption. The largest negative effect was observed for the E465/E6 ratio (−0.829, *p* = 0.0005), indicating that humic acids hinder the effective sorption of dimethoate. The H/C ratio (−0.364, *p* = 0.0013) also showed a negative impact on sorption, implying that an increased H/C ratio reduces the stability of organic matter in the sorption of dimethoate. In addition, the (N+O)/C ratio (−0.272, *p* = 0.0313), which reflects the polarity of soil organic matter, was negatively correlated with sorption. This suggests that increased polarity, possibly due to competition between dimethoate and other N- and O-containing compounds for the same sorption sites, reduces the ability of the soil to retain the pesticide. K^+^ content (−0.382, *p* = 0.0025) was also a negative predictor, suggesting that high K^+^ content may inhibit dimethoate sorption, possibly through competitive binding for available sites. Somewhat surprisingly, the ratios of fulvic and humic acid, although included in the model, did not reach statistical significance, suggesting that these specific soil organic matter components do not directly influence dimethoate sorption in this context.

The dependent variable in the study on the desorption of dimethoate, $K_{F}^{\mathrm{des}}$, is the ability of the soil to retain dimethoate after desorption. The desorption model, as shown in Table 9, provided an even better fit than the sorption model, with *R*^2^ = 0.9998 and adjusted *R*^2^ = 0.9995, explaining almost all the variation in the sorbed fraction of dimethoate remaining. The high F-value of 3277.58 and *p*-value of less than 0.0001 confirm the strong statistical significance of the model and the high correlation between the selected variables and the desorption processes. Among the predictors, TOC had the most significant positive influence on $K_{F}^{\mathrm{des}}$ with a coefficient of 1.278 (*p* = 0.0018), followed by the (N+O)/C ratio (1.594, *p* = 0.003). These results indicate that organic matter, and especially oxygen-containing functional groups increase dimethoate retention by forming additional binding sites and thus reducing desorption. Additional positive effects were observed for the C/N ratio (0.630, *p* = 0.0004), E465/E665 (0.259, *p* = 0.0001), and fulvic acid content, all suggesting that N-rich organic matter and fulvic acids enable tighter binding of the pesticide and reduce its desorption rate. For mineral soil properties, clay content had a positive effect on dimethoate retention with a coefficient of 0.187 (*p* = 0.0475), supporting the notion that higher clay content improves binding capacity. K^+^ also showed a positive effect (0.070, *p* = 0.0046), although its small coefficient suggests that it plays a relatively minor role in dimethoate retention, possibly through specific physicochemical interactions. In contrast, the H/C and S/C ratios were included in the model but show no significant influence, indicating that these factors play a lesser role in the desorption process.

### 3.8. Dimethoate Sorption/Desorption Dynamics: Interplay Between Organic Matter, Clay, and Soil Mineralogy

The results of this study emphasize the crucial role of soil physicochemical properties in determining the sorption and desorption behavior of dimethoate, a widely used organophosphate pesticide. The physicochemical properties of the soils showed significant differences in the sorption and desorption capacity of dimethoate. Soil organic matter played a crucial role in influencing the behavior of dimethoate. Soil S1 with a higher content of TOC showed better sorption properties. These soils exhibited better structure and fertility, suggesting that higher organic matter favors stronger interactions with dimethoate. In contrast, soils with a lower TOC content, such as S4, exhibited poorer sorption due to their less favorable organic matter composition and less flexible structures in the glassy phase. The differences between the H/C and O/C ratios indicate that soils with a higher H/C ratio—for example, S5—contain a greater proportion of degradable aliphatic compounds, while soils with a higher O/C ratio, such as S4, are more strongly associated with polar dimethoate due to their higher content of oxygen-containing functional groups. These results confirm previous studies emphasizing the importance of H/C and O/C ratios for soil-pesticide interactions [93,94].

The sorption of dimethoate to the organic phase of the soil probably occurs by two parallel processes: dissolution into the solid phase and filling of void [95]. Dissolution in extended regions of the soil organic phase dominates in soils with a higher proportion of flexible organic matter, where humus structures remain flexible and are exposed to thermal vibrations. These vibrations create temporary sorption sites that allow dimethoate to bind without competitive interactions at lower concentration [96]. This behavior of organic matter is characteristic of the rubbery phase, which enables the linear and non-competitive sorption of dimethoate at low concentrations. The rubbery phase allows dynamic and flexible binding of dimethoate within the organic matter, without interference with other molecules. In contrast, in soils with lower organic matter, such as soil S4, the sorption of dimethoate follows a different mechanism and mainly takes place in the glassy phase. Here, the sorption sites are less flexible and smaller, resulting in competing interactions. Under these conditions, the binding of pesticides is more sensitive to higher concentrations of pesticides and exogenous factors [95]. Studies by Gunasekara and Xing [97], indicate that the flexible aliphatic compounds of soil in the rubbery phase can stabilize pesticides such as dimethoate more effectively via van der Waals interactions, thus reducing their mobility. Conversely, aromatic compounds typical of a glassy phase provide less effective π–π interactions, making them weaker at retaining dimethoate compared to the van der Waals forces provided by the rubbery phase. Our results clearly show that dimethoate interacts better with flexible aliphatic compounds such as those found in soil S5. Furthermore, dimethoate showed strong interactions with negatively charged components of soil organic matter, especially with carboxyl and phenolic groups. This is consistent with the results of Meftaul et al. [53], who emphasized the importance of both polar and apolar pesticide regions for interactions with organic matter. Dimethoate has one hydrogen bond donor and five acceptor sites, which enables it to form stable interactions with hydrophilic soil components such as organic matter and clay. Its topological polar surface area of 105 Å^2^, enables high interaction with polar soil components and improves sorption. This is consistent with the previous findings [53,57,93], that such hydrogen bonding significantly increases the stability of pesticides and reduces mobility and leaching potential. FTIR analyses of certain Australian soils revealed that organic matter contributed carboxyl and alkyl groups, which play an important role in polarity, chemical reactivity, and cation exchange. According to Meftaul et al. [53], these same groups increase the sorption of dimethoate in urban soils, emphasizing organic matter as an important factor in the retention of pesticides. In support, Eissa et al. [93] observed an increase in surface area due to micropores in biochar, which enhanced the sorption of dimethoate. Biochar has hydrophobic sites that retain non-polar pesticides, and various functional groups (-OH, -COOH, -CH_3_ -Ph-OH) that enhance van der Waals forces and hydrogen bonding, thereby improving dimethoate retention. According to Broznić et al. [51], the organic matter content in the soil is the dominant factor for the sorption of dimethoate if it is present in sufficient quantities. Soils with high organic matter content, such as S1, showed a higher sorption capacity, which is consistent with the results of other studies. On the other hand, the lower TOC content in soil S4 did not lead to a significantly weaker sorption of dimethoate. This indicates that, in addition to organic matter, inorganic soil components also play an important role in the sorption process.

The texture of the investigated soils ranged from sandy loam to clayey compositions. The high clay content in soil S4 indicates a balance between water retention and drainage, while the sandy loam texture of soil S2 suggests a rather well-draining nature with medium nutrient retention capacity. The clay content therefore played an important role in the sorption of dimethoate. Soils with a higher clay content, such as S4 (40.58%), had a higher sorption capacity. In this soil, dimethoate could be retained by hydrogen bonds formed between the C=O group of pesticide and the -Al-OH or -Si-OH groups of the clay surface. The weakly negatively charged components of the pesticide can potentially interact electrostatically with Mg^2+^ ions due to the high concentrations of Mg^2+^ ions. As a final mechanism for the stabilization of dimethoate in soil S4, we hypothesize that the -P=S group of the pesticide forms complexes with transition metals such as Fe^3+^. In contrast, soils S2 and S3 with a low clay content (less than 21%), showed a weaker sorption potential, leading to a higher probability of dimethoate leaching. In particular, soil S2 is dominated by the sand fraction, which has a weak binding affinity for dimethoate and a small reactive surface area for binding. Similarly, the value 1/*n* = 1.143 indicates a weak affinity for the sorption of dimethoate, which means that the pesticide is mainly located in the aqueous phase of the soil. In soil S3, a representative of reddish soils, the presence of hematite reduces the availability of -Al-OH and -Si-OH groups on the clay to bind dimethoate. As a result, hydrogen bonding and electrostatic interactions are expected to be minimized. In the S1 and S5 soils with moderate sorption, the stabilization of dimethoate was also influenced by other factors, such as CEC and organic matter content, which affected the sorption capacity through competing processes. We hypothesize that the binding of dimethoate in these soils was achieved by a combination of electrostatic interactions with metal cations in the soil, hydrogen bonding, and complexation of the -P=S group of dimethoate with Fe^3+^ ions. These results support previous studies showing that negatively charged groups on clays particles contribute to the stabilization of dimethoate [53].

Overall, increasing soil organic matter content did not always increase the amount of dimethoate sorbed, which is consistent with trends for other highly polar pesticides [57,88,97]. When organic matter was below 5%, other variables, such as the structure of the pesticide, availability of functional groups, inorganic soil constituents, and general soil properties had a greater influence [51,98,99]. An important inorganic factor influencing the sorption of dimethoate is the mineral phase of the soil. Based on these results, we can confirm the findings of authors such as Calvet et al. [77], who observed that all molecules, except hydrophobic ones, are able to sorb to clay surfaces. Furthermore, Sheng et al. [78] reported that clays sorbed pesticides, such as dichlobenil and carbaryl more strongly compared to organic matter, confirming our observations in this study regarding the role of clay in the stabilization of dimethoate in soil S1. Numerous studies have demonstrated the importance of clay in the sorption mechanism of pesticides [51,54,55,56,76,78]. In addition, the studies of Gunasekara and Xing [96] show how interaction between the mineral phase and the organic matrix leads to the formation of a “condensed phase”, that affects the sorption of pesticides, including dimethoate. This condensed phase is formed through organic–inorganic interaction, creating stable structures that improve pesticide retention, thus reducing mobility and consequently minimizing the risk of groundwater contamination. In our study, organic matter plays only a minor role in the retention of dimethoate, while other inorganic components—such as calcium concentration [100], cation exchange capacity [57,101]—appear to have greater influence. In addition, dimethoate was more stabilized in soils with higher metal ion concentrations. This is supported by Meftaul et al. [53], who found that metal ions play the crucial role in stabilizing pesticides in soils. These findings have important implications for agricultural practices, particularly in areas with different soil types, and highlight the need for careful management of pesticide use to minimize environmental contamination.

## 4. Conclusions

The results of this study highlight the complex interaction between soil properties and dimethoate, a polluting pesticide, and focus on the role of soil organic matter and mineral properties in regulating stability, mobility, and contamination risk. Organic matter, especially TOC, plays an important role in the stability of dimethoate. A high TOC content creates a large number of binding sites for sorption, which reduces the mobility of dimethoate and thus its leaching potential. However, it is likely that the acidic oxygenated groups, O/C, and (N+O)/C weaken the binding of dimethoate and increase its mobility and the risk of groundwater contamination. The dual polar and non-polar properties of dimethoate lead to stronger interactions with the rubbery phase of the soil. In the aliphatic, rubbery phase, these interactions stabilize dimethoate through van der Waals forces, reducing its mobility and increasing its stability in the soil. In contrast, the aromatic-rich glassy phase supports weaker π–π interactions between dimethoate and aromatic rings. The polar phosphate group in dimethoate enhances bonding to the rubbery phase, which consists mainly of aliphatic compounds that may contain functional groups such as hydroxyl (-OH) and carboxyl (-COOH) groups that could enable hydrogen bonding. This difference in interactions between the rubbery and glassy soil phases has a great influence on the stability and mobility of dimethoate. The distribution between these phases depends on the ratio of aliphatic and aromatic compounds in the soil organic matter, which is indicated by the H/C and O/C ratios. Soils with a higher H/C ratio contain more aliphatic compounds, while the rubbery phase is better suited to bind dimethoate. On the other hand, the glassy phase in soils with higher O/C ratios may allow weaker interactions due to the higher content of aromatic compounds. The second most important factor influencing the sorption of dimethoate is therefore the soil texture, with a high clay content being particularly important. A high clay content increases the sorption capacity due to the large specific surface area and the cation exchange capacity. A high content of minerals such as magnesium, potassium, and calcium cations strongly influenced the stabilization of dimethoate in the mineral composition of the investigated soils. Magnesium and potassium are positively correlated with dimethoate stabilization, while calcium, especially under alkaline conditions, can reduce sorption efficiency by influencing soil structure. The study also emphasizes the importance of hysteresis and thermodynamic processes for the dynamics of sorption and desorption. Soils with high clay content have high hysteresis coefficients, indicating poor desorption of dimethoate and increased stability in these soils, reducing mobility and consequently the risk of leaching to groundwater. Negative Gibbs free energy values also confirm the spontaneous sorption of dimethoate, supporting stability in soil with high concentrations of organic compounds.

All these results show the importance of proper agriculture management to reduce the risk of groundwater leaching through the use of dimethoate. Increasing the organic matter and clay content of the soil and managing the ratio of humic-to-fulvic acid appropriately will significantly reduce the mobility of dimethoate and the associated ecological risk. In addition, the management of cations such as calcium, magnesium, and potassium can improve the stability of dimethoate. To understand the interactions between organic matter, mineral soil properties and pesticides, it is therefore necessary to analyze sorption–desorption dynamics, taking into account hysteresis and thermodynamic processes. This ensures the sustainable agricultural use of dimethoate while minimizing its environmental impact.

## Figures and Tables

**Table 2:** Physicochemical properties of the investigated soil samples.

Soil Properties	Soil				
	S1	S2	S3	S4	S5
Location	Grobnik	Matulji 1	Matulji 2	Varaždin	Otočac
GCS ^(a)^	45°20′53″ N 14°30′04″ E	45°21′27″ N 14°18′20″ E	45°21′28″ N 14°18′19″ E	46°17′17″ N 16°44′15″ E	44°56′31″ N 15°09′08″ E
Textural classes	clay loam	sandy loam	sandy clay loam	clay	sandy clay loam
pH (H_2_O) ^(b)^	7.03 (±0.19)	7.10 (±0.09)	7.11 (±0.04)	7.01 (±0.08)	6.73 (±0.09)
pH (CaCl_2_) ^(b)^	6.57 (±0.03)	6.44 (±0.00)	6.42 (±0.02)	6.54 (±0.01)	6.21 (±0.01)
HA (cmol/kg) ^(c)^	13.14 (±0.68)	11.06 (±1.13)	13.29 (±1.13)	3.74 (±0.26)	27.51 (±0.52)
CEC ^(d)^ (cmol/kg)	74.39 (±17.19)	91.32 (±1.22)	79.83 (±8.88)	89.37 (±8.87)	66.12 (±3.48)
Clay (%)	35.46 (±0.58)	17.35 (±0.31)	20.50 (±0.74)	40.58 (±0.61)	28.14 (±0.46)
Ca^2+^ (mg/100 g)	109.70 (±3.10)	333.43 (±2.97)	375.17 (±69.42)	224.10 (±39.19)	186.50 (±4.07)
Mg^2+^ (mg/100 g)	519.10 (±172.30)	577.57 (±15.35)	460.80 (±88.00)	785.60 (±111.70)	339.00 (±26.67)
Na^+^ (mg/100 g)	38.55 (±0.48)	37.89 (±1.55)	32.02 (±5.54)	37.83 (±5.58)	34.42 (±3.63)
K^+^ (mg/100 g)	446.30 (±118.70)	565.97 (±1.85)	333.47 (±3.45)	320.47 (±39.95)	324.90 (±51.57)
TOC ^(e)^ (%)	5.75 (±0.20)	3.68 (±0.12)	5.20 (±0.08)	1.96 (±0.09)	3.68 (±0.06)
C_oxHa_^(f)^ (%)	0.083 (±0.001)	0.133 (±0.054)	0.069 (±0.054)	0.086 (±0.004)	0.118 (±0.008)
C_oxFa_^(g)^ (%)	0.257 (±0.025)	0.259 (±0.095)	0.254 (±0.045)	0.160 (±0.021)	0.312 (±0.021)
N (%)	0.4665 (±0.0061)	0.2778 (±0.0037)	0.4135 (±0.0027)	0.2003 (±0.0042)	0.3062 (±0.0068)
C (%)	4.722 (±0.077)	3.239 (±0.132)	4.449 (±0.022)	1.580 (±0.012)	2.965 (±0.011)
H (%)	1.662 (±0.013)	1.028 (±0.017)	1.139 (±0.018)	0.473 (±0.07)	1.093 (±0.017)
S (%)	0.039 (±0.0035)	0.035 (±0.0060)	0.035 (±0.0040)	0.012 (±0.0031)	0.019 (±0.0020)
O (%)	93.111 (±0.089)	95.421 (±0.122)	93.965 (±0.037)	97.735 (±0.018)	95.618 (±0.013)
ratio H/C	4.197 (±0.052)	3.778 (±0.193)	3.049 (±0.039)	3.570 (±0.027)	4.391 (±0.084)
ratio C/N	11.804 (±0.053)	13.594 (±0.457)	12.548 (±0.120)	9.204 (±0.148)	11.296 (±0.292)
ratio S/C	0.003 (±0.000)	0.004 (±0.001)	0.003 (±0.000)	0.003 (±0.001)	0.002 (± 0.000)
ratio O/C	14.802 (±0.868)	22.115 (±0.695)	15.855 (±1.278)	46.436 (±1.141)	24.209 (±0.867)
ratio (N+O)/C	14.891 (±0.254)	22.217 (±0.949)	15.935 (±0.084)	46.546 (±0.348)	24.303 (±0.090)
ratio E465/E665	4.73 (±0.02)	5.07 (±0.11)	6.25 (±0.44)	4.10 (±0.27)	5.09 (±0.29)

^(a)^—Geographic Coordinate System; ^(b)^—measured in soil + H_2_O or 0.01 M calcium chloride mixture (1:2.5 *w*/*v*); ^(c)^—hydrolytic acidity; ^(d)^—cation exchange capacity; ^(e)^—total organic carbon content; ^(f)^—humic acids content; ^(g)^—fulvic acids content.

**Table 3:** Fitted and statistical parameters for the sorption of dimethoate in Croatian soils (S1–S5) obtained by the Freundlich, Langmuir, and Temkin isotherm models. Results are given as mean ± standard deviation.

Fitted/Statistical Parameter	S1	S2	S3	S4	S5
Freundlich isotherm model					
$K_{F}^{\mathrm{sor}}$ ^(a)^ (mg/kg) (mg/L)^1/n^	3.069 (±0.743)	1.360 (±0.173)	1.730 (±0.561)	4.701 (±0.191)	2.159 (±0.517)
1/n ^(b)^	0.967 (±0.093)	1.143 (±0.019)	1.215 (±0.112)	0.799 (±0.001)	1.166 (±0.084)
*R* ^2 (c)^	0.9681	0.9305	0.9739	0.9581	0.9773
SRMSE ^(d)^	0.1093	0.2025	0.1027	0.1732	0.0823
err-% ^(e)^	8.69	16.10	8.17	13.77	6.55
m ^(f)^	4 ($\chi_{\mathrm{tab}\;}^{2}=$ 9.49 at *p* = 0.05)				
Langmuir isotherm model					
$\;K_{L}^{\mathrm{sor}}$ ^(g)^ (L/kg)	0.0125 (±0.0148)	0.0229 (±0.0054)	−0.0138 (±0.0094)	0.0263 (±0.0156)	0.0017 (±0.0125)
$q_{\max}^{\mathrm{sor}}$ ^(h)^ (mg/kg)	788.91 (±904.61)	104.52 (±28.97)	−215.15 (±183.39)	174.61 (±74.47)	−17.28 (±480.64)
*R* ^2^	0.9428	0.7940	0.9730	0.9753	0.9388
SRMSE	0.2975	0.9161	0.3635	0.2549	0.3407
err-%	23.66	72.84	29.05	20.27	27.09
m	4 ($\chi_{\mathrm{tab}\;}^{2}=$ 9.49 at *p* = 0.05)				
Temkin isotherm model					
$K_{T}^{\mathrm{sor}}$ ^(i)^ (L/mg)	0.2488 (±0.0374)	0.1836 (±0.004)	0.2119 (±0.0161)	0.3193 (±0.0249)	0.2200 (±0.0145)
$B_{1}^{\mathrm{sor}}$ ^(j)^	51.29 (±10.79)	56.05 (±2.21)	68.24 (±5.94)	37.59 (±3.82)	71.98 (±5.20)
*R* ^2^	0.8300	0.6968	0.9080	0.8727	0.8303
SRMSE	0.3677	0.5135	0.2970	0.3059	0.4104
err-%	29.24	40.83	23.62	25.91	32.63
m	4 ($\chi_{\mathrm{tab}\;}^{2}=$ 9.49 at *p* = 0.05)				

^(a)^—Freundlich’s sorption coefficient; ^(b)^—nonlinearity coefficient; ^(c)^—coefficient of multiple determination; ^(d)^—scaled root mean squared error; ^(e)^—minimum error level of *χ*^2^ test; ^(f)^—degrees of freedom = number of measurements-number of model parameters; ^(g)^—Langmuir constant; ^(h)^—maximum amount of dimethoate sorbed by the soil; ^(i)^—equilibrium binding constant; ^(j)^—constant related to the heat of sorption.

**Table 4:** Fitted and statistical parameters for the desorption of dimethoate in Croatian soils (S1–S5), determined with the isotherm models of Freundlich, Langmuir, and Temkin. The results are given as mean ± standard deviation.

Fitted/Statistical Parameter	S1	S2	S3	S4	S5
Freundlich isotherm model					
$K_{F}^{\mathrm{des}}$ ^(a)^ (mg/kg) (mg/L)^1/n^	6.695 (±0.451)	3.482 (±0.019)	5.725 (±0.075)	9.096 (±0.077)	6.238 (±0.051)
1/n ^(b)^	0.866 (±0.004)	0.911 (±0.006)	0.863 (±0.008)	0.809 (±0.004)	0.872 (±0.001)
*R* ^2 (c)^	0.9912	0.9989	0.9941	0.9924	0.9948
SRMSE ^(d)^	0.0856	0.0689	0.0679	0.0720	0. 0468
err-% ^(e)^	6.80	5.48	5.40	5.73	3.72
m ^(f)^	4 ($\chi_{\mathrm{tab}\;}^{2}=$ 9.49 at *p* = 0.05)				
Langmuir isotherm model					
$\;K_{L}^{\mathrm{des}}$ ^(g)^ (L/kg)	0.0519 (±0.0233)	0.0200 (±0.0007)	0.0313 (±0.0022)	0.0760 (±0.0025)	0.0335 (±0.0061)
$q_{\max}^{\mathrm{des}}$ ^(h)^ (mg/kg)	149.06 (±66.49)	174.03 (±6.42)	183.87 (±11.93)	127.44 (±3.44)	220.02 (±10.26)
*R* ^2^	0.9779	0.9691	0.9738	0.9802	0.9523
SRMSE	0.2187	0.2311	0.2201	0.1794	0.2969
err-%	17.39	18.38	17.50	14.27	23.62
m	4 ($\chi_{\mathrm{tab}\;}^{2}=$ 9.49 at *p* = 0.05)				
Temkin isotherm model					
$K_{T}^{\mathrm{des}}$ ^(i)^ (L/mg)	1.0767 (±0.0970)	0.8934 (±0.0934)	1.1334 (±0.0730)	1.3799 (±0.2077)	0.9715 (±0.0403)
$B_{1}^{\mathrm{des}}$ ^(j)^	22.76 (±4.07)	16.85 (±1.22)	20.84 (±0.01)	21.51 (±2.48)	25.51 (±0.27)
*R* ^2^	0.9209	0.8733	0.9270	0.9463	0.9159
SRMSE	0.3407	0.3984	0.3321	0.3146	0.3584
err-%	27.09	31.68	26.40	25.02	28.50
m	4 ($\chi_{\mathrm{tab}\;}^{2}=$7.81 at *p* = 0.05)				

^(a)^—Freundlich’s desorption coefficient; ^(b)^— nonlinearity coefficient; ^(c)^—coefficient of multiple determination; ^(d)^— scaled root mean squared error; ^(e)^—minimum error level of *χ*^2^ test; ^(f)^ —degrees of freedom = number of measurements-number of model parameters; ^(g)^— Langmuir constant; ^(h)^—maximum amount of dimethoate remain sorbed by the soil; ^(i)^—equilibrium binding constant; ^(j)^— constant related to the heat of desorption.

**Table 5:** Physicochemical and thermodynamic parameters (*K*_OC_, ΔG, *H*, and *λ* values) for the sorption and desorption of dimethoate in Croatian soils (S1–S5). The results are given as mean ± standard deviation.

Parameters	S1	S2	S3	S4	S5
*K*_OC_ ^(a)^ (L/kg)	53.63 (±14.76)	37.09 (±5.91)	33.36 (±11.33)	240.49 (±1.53)	58.57 (±13.14)
ΔG ^(b)^ (kJ/mol)	−9.66 (±0.68)	−8.79 (±0.39)	−8.48 (±0.84)	−13.36 (±0.02)	−9.89 (±0.55)
*H* ^(c)^	0.899 (±0.090)	0.797 (±0.008)	0.713 (±0.059)	1.012 (±0.003)	0.750 (±0.054)
*λ* ^(d)^	0.055 (±0.052)	0.121 (±0.006)	0.189 (±0.055)	−0.005 (±0.002)	0.157 (±0.045)

^(a)^—organic carbon partition coefficient; ^(b)^—Gibbs free energy; ^(c), (d)^—hysteresis coefficients.

**Table 6:** Analysis of matrix correlations of soil properties and Freundlich model parameters for the sorption and desorption of dimethoate in soils (S1–S5). Statistically significant correlations (*p* < 0.05) are shown in bold.

Variable	$K_{F}^{sor\;\left(f\right)}$	$1/n^{sor}$ ^(g)^	$K_{F}^{des\;\left(h\right)}$	$1/n^{des}$ ^(i)^	*K*_OC_ ^(j)^	∆G ^(k)^	*H* ^(l)^	*λ* ^(m)^
pH (CaCl_2_)	0.42	**−0.59** (*p* = 0.021)	0.21	−0.17	0.28	−0.15	**0.58** (*p* = 0.024)	**−0.61** (*p* = 0.017)
HA ^(a)^	−0.49	**0.61** (*p* = 0.016)	−0.30	0.34	**−0.57** (*p* = 0.026)	0.35	**−0.59** (*p* = 0.020)	**0.62** (*p* = 0.014)
CEC ^(b)^	0.01	−0.14	−0.04	0.02	0.28	−0.05	0.12	−0.19
Clay	**0.92** (*p* < 0.001)	**−0.85** (*p* < 0.001)	**0.92** (*p* < 0.001)	**−0.80** (*p* < 0.001)	**0.76** (*p* = 0.001)	**−0.82** (*p* < 0.001)	**0.82** (*p* < 0.001)	**−0.76** (*p* = 0.001)
TOC ^(c)^	−0.48	0.44	−0.40	0.42	**−0.78** (*p* = 0.001)	**0.75** (*p* = 0.001)	−0.45	0.46
C_oxHa_^(d)^	−0.10	−0.11	−0.21	0.30	−0.01	0.12	0.06	−0.09
C_oxFa_ ^(e)^	**−0.58** (*p* = 0.022)	**0.61** (*p* = 0.017)	**−0.52** (*p* = 0.045)	**0.53** (*p* = 0.042)	**−0.68** (*p* = 0.005)	0.51	**−0.59** (*p* = 0.022)	**0.57** (*p* = 0.027)
N	0.33	0.32	−0.25	0.27	**−0.68** (*p* = 0.006)	**0.63** (*p* = 0.011)	−0.33	0.36
C	**−0.52** (*p* = 0.045)	0.47	−0.47	0.47	**−0.80** (*p* < 0.001)	**0.79** (*p* < 0.001)	−0.47	0.48
H	−0.41	0.34	−0.40	0.50	**−0.76** (*p* = 0.001)	**0.65** (*p* = 0.009)	−0.31	0.32
S	**−0.60** (*p* = 0.018)	0.44	**−0.64** (*p* = 0.010)	**0.66** (*p* = 0.008)	**−0.77** (*p* = 0.001)	**0.83** (*p* < 0.001)	−0.42	0.39
O	0.50	−0.44	0.45	−0.47	**0.80** (*p* < 0.001)	**−0.76** (*p* = 0.001)	0.43	−0.44
Mg	**0.52** (*p* = 0.049)	**−0.61** (*p* = 0.015)	0.43	−0.41	**0.68** (*p* = 0.005)	−0.51	**0.58** (*p* = 0.023)	**−0.61** (*p* = 0.015)
K	−0.43	0.17	**−0.62** (*p* = 0.014)	**0.68** (*p* = 0.005)	−0.40	0.45	−0.12	0.03
Na	0.32	**−0.52** (*p* = 0.049)	0.08	−0.02	0.25	−0.28	0.51	**−0.57** (*p* = 0.028)
Ca	−0.30	0.23	−0.36	0.20	−0.07	0.34	−0.24	0.20
Ratio E465/E665	**−0.79** (*p* < 0.001)	**0.86** (*p* < 0.001)	**−0.57** (*p* = 0.026)	0.43	**−0.73** (*p* = 0.002)	**0.81** (*p* < 0.001)	**−0.88** (*p* = 0.002)	**0.90** (*p* < 0.001)
Ratio H/C	0.04	−0.08	−0.01	0.21	−0.14	−0.11	0.15	−0.14
Ratio C/N	**−0.89** (*p* < 0.001)	**0.74** (*p* = 0.002)	**−0.96** (*p* < 0.001)	**0.91** (*p* < 0.001)	**−0.89** (*p* < 0.001)	**0.92** (*p* < 0.001)	**−0.70** (*p* = 0.004)	**0.64** (*p* = 0.011)
Ratio S/C	−0.36	0.14	**−0.52** (*p* = 0.046)	**0.56** (*p* = 0.029)	−0.25	0.40	−0.10	0.03
Ratio O/C	**0.73** (*p* = 0.002)	**−0.67** (*p* = 0.006)	**0.68** (*p* = 0.006)	**−0.68** (*p* = 0.006)	**0.95** (*p* < 0.001)	**−0.90** (*p* < 0.001)	**0.65** (*p* = 0.008)	**−0.64** (*p* = 0.010)
Ratio (N + O)/C	**0.73** (*p* = 0.002)	**−0.67** (*p* = 0.006)	**0.68** (*p* = 0.006)	**−0.68** (*p* = 0.006)	**0.95** (*p* < 0.001)	**−0.90** (*p* < 0.001)	**0.65** (*p* = 0.008)	**−0.64** (*p* = 0.010)

^(a)^—hydrolytic acidity; ^(b)^—cation exchange capacity; ^(c)^—total organic carbon content; ^(d)^—humic acids content; ^(e)^—fulvic acids content; ^(f), (h)^—Freundlich’s sorption and desorption coefficient; ^(g), (i)^—nonlinearity coefficient; ^(j)^—organic carbon partition coefficient; ^(k)^—Gibbs free energy; ^(l), (m)^—hysteresis coefficients.

**Table 7:** Eigenvalues of Principal Component Analysis (PCA), variance contributions, and loadings for physicochemical soil properties and Freundlich sorption/desorption parameters of dimethoate in soils (S1–S5).

Principal Component	PC 1	PC 2	PC 3	PC 4
Eigenvalue	15.25	5.11	4.19	2.85
% Total variance	50.84	17.05	13.96	9.51
Cumulative %	50.84	67.89	81.85	91.36
Loadings				
pH	−0.261	0.619	0.675	−0.232
HA ^(a)^	0.534	−0.730	−0.211	0.321
CEC ^(b)^	−0.270	0.850	−0.182	−0.017
Clay	−0.772	−0.298	0.501	−0.014
TOC ^(c)^	0.803	−0.045	0.545	−0.208
C_oxHa_^(d)^	−0.031	0.427	−0.129	0.524
C_oxFa_^(e)^	0.667	−0.479	−0.115	0.242
N	0.702	−0.133	0.634	−0.272
C	0.831	0.007	0.502	−0.234
H	0.741	−0.140	0.636	0.119
S	0.776	0.444	0.404	−0.019
O	−0.816	0.034	−0.551	0.154
Mg	−0.679	0.082	0.638	−0.052
K	0.339	0.133	0.673	0.537
Na	−0.312	0.251	0.412	0.509
Ca	0.156	0.756	−0.305	−0.518
ratio E465/E665	0.799	0.002	−0.314	−0.477
ratio H/C	0.027	−0.450	0.256	0.804
ratio C/N	−0.933	−0.262	0.055	−0.120
ratio S/C	0.238	0.803	−0.039	0.285
ratio O/C	−0.952	0.041	−0.291	0.034
ratio (N+O)/C	−0.952	0.041	−0.291	0.034
*K*_F_^sor (f)^	−0.894	−0.154	0.388	−0.105
1/*n*^sor (g)^	0.851	−0.068	−0.490	−0.051
*K*_F_^des (h)^	−0.808	−0.337	0.263	−0.291
1/*n*^des (i)^	0.780	0.310	−0.144	0.457
*K*_OC_ ^(j)^	−0.991	0.011	−0.014	−0.115
∆G ^(k)^	0.963	0.226	−0.053	−0.032
*λ* ^(l)^	−0.827	0.077	0.488	0.125
*H* ^(m)^	0.807	−0.156	−0.472	−0.182

^(a)^—hydrolytic acidity; ^(b)^—cation exchange capacity; ^(c)^—total organic carbon; ^(d)^—carbon of humic acids; ^(e)^—carbon of fulvic acids; ^(f), (g), (h), (i)^—parameters obtained by modelling with Freundlich model; ^(j)^—organic carbon partition coefficient; ^(k)^—molar free Gibbs energy; ^(l), (m)^—hysteresis coefficients.

**Table 8:** Evaluation of dimethoate sorption in soils (S1–S5) using Multiple Regression Analysis with predictor variables and statistical coefficients. Statistically significant correlations (*p* < 0.05) are in bold.

**Statistic**	**Value**			
*R* ^2^	0.9891			
Adjusted *R*^2^	0.9782			
F-value	90.56			
*p*-value (F)	<0.0001			
Std. Err. of Estimate	0.1886			
Predictor	Coefficient b*	Std. Err. of b*	T value (t (7))	*p*-value
Clay	**0.364**	**0.107**	**3.40**	**0.0115**
Ratio (N+O)/C	**−** **0.272**	**0.101**	**−** **2.68**	**0.0313**
Ratio H/C	**−** **0.364**	**0.071**	**−** **5.15**	**0.0013**
Ratio E465/E665	**−** **0.829**	**0.135**	**−** **6.12**	**0.0005**
K	**−** **0.382**	**0.083**	**−** **4.60**	**0.0025**
C_oxFa_^(a)^	−0.093	0.076	−1.22	0.2614
C_oxHa_^(b)^	0.057	0.055	1.04	0.3335

^(a)^–carbon of fulvic acids; ^(b)^–carbon of humic acids; b*–the estimated effect of the predictor variable on the dependent variable, with the asterisk indicating statistical significance.

**Table 9:** Evaluation of dimethoate desorption in soils (S1–S5) using Multiple Regression Analysis including predictor variables and statistical coefficients. Statistically significant correlations (*p* < 0.05) are in bold.

**Statistic**	**Value**			
*R* ^2^	0.9998			
Adjusted *R*^2^	0.9995			
F-value	3277.58			
*p*-value (F)	<0.0001			
Std. Err. of Estimate	0.0414			
Predictor	Coefficient b*	Std. Err. of b*	T value (t (7))	*p*-Value
Clay	**0.187**	**0.071**	**2.61**	**0.0475**
TOC	**1.278**	**0.211**	**6.05**	**0.0018**
C_oxFa_^(a)^	**0.289**	**0.021**	**13.74**	**<0.0001**
Ratio (N+O)/C	**1.594**	**0.297**	**5.38**	**0.0030**
Ratio H/C	0.030	0.033	0.90	0.4109
Ratio E465/E665	**0.259**	**0.021**	**12.34**	**0.0001**
Ratio C/N	**0.630**	**0.075**	**8.38**	**0.0004**
Ratio S/C	−0.024	0.020	−1.19	0.2876
K	**0.070**	**0.014**	**4.87**	**0.0046**

^(a)^—carbon of fulvic acids; b*—the estimated effect of the predictor variable on the dependent variable, with the asterisk indicating statistical significance.

## Data Availability

The data supporting the findings of this study are available from the corresponding author upon reasonable request.
